# Applying a modified systematic review and integrated assessment framework (SYRINA) – a case study on triphenyl phosphate†

**DOI:** 10.1039/d3em00353a

**Published:** 2023-12-26

**Authors:** Thuy T. Bui, Jenny Aasa, Khaled Abass, Marlene Ågerstrand, Anna Beronius, Mafalda Castro, Laura Escrivá, Audrey Galizia, Anda Gliga, Oskar Karlsson, Paul Whaley, Erin Yost, Christina Rudén

**Affiliations:** a Department of Environmental Science, Stockholm University Sweden christina.ruden@aces.su.se; b Swedish Food Agency Sweden; c Department of Environmental Health Sciences, College of Health Sciences, University of Sharjah United Arab Emirates; d Sharjah Institute for Medical Research (SIMR), University of Sharjah United Arab Emirates; e Research Unit of Biomedicine and Internal Medicine, Faculty of Medicine, University of Oulu Finland; f Institute of Environmental Medicine, Karolinska Institutet Sweden; g Section for Environmental Chemistry and Physics, University of Copenhagen Denmark; h Laboratory of Food Chemistry and Toxicology, Faculty of Pharmacy, University of Valencia Spain; i United States Environmental Protection Agency, Center for Public Health and Environmental Assessment USA; j Science for Life Laboratory, Department of Environmental Science, Stockholm University Sweden; k Lancaster Environment Centre, Lancaster University UK

## Abstract

This work presents a case study in applying a systematic review framework (SYRINA) to the identification of chemicals as endocrine disruptors. The suitability and performance of the framework is tested with regard to the widely accepted World Health Organization definition of an endocrine disruptor (ED). The endocrine disrupting potential of triphenyl phosphate (TPP), a well-studied flame retardant reported to exhibit various endocrine related effects was assessed. We followed the 7 steps of the SYRINA framework, articulating the research objective *via* Populations, Exposures, Comparators, Outcomes (PECO) statements, performed literature search and screening, conducted study evaluation, performed data extraction and summarized and integrated the evidence. Overall, 66 studies, consisting of *in vivo*, *in vitro* and epidemiological data, were included. We concluded that triphenyl phosphate could be identified as an ED based on metabolic disruption and reproductive function. We found that the tools used in this case study and the optimizations performed on the framework were suitable to assess properties of EDs. A number of challenges and areas for methodological development in systematic appraisal of evidence relating to endocrine disrupting potential were identified; significant time and effort were needed for the analysis of *in vitro* mechanistic data in this case study, thus increasing the workload and time needed to perform the systematic review process. Further research and development of this framework with regards to grey literature (non-peer-reviewed literature) search, harmonization of study evaluation methods, more consistent evidence integration approaches and a pre-defined method to assess links between adverse effect and endocrine activity are recommended. It would also be advantageous to conduct more case studies for a chemical with less data than TPP.

## Abstract 2

This work presents a case study in applying a systematic review framework (SYRINA) to the identification of chemicals as endocrine disruptors.

## Graphical abstract

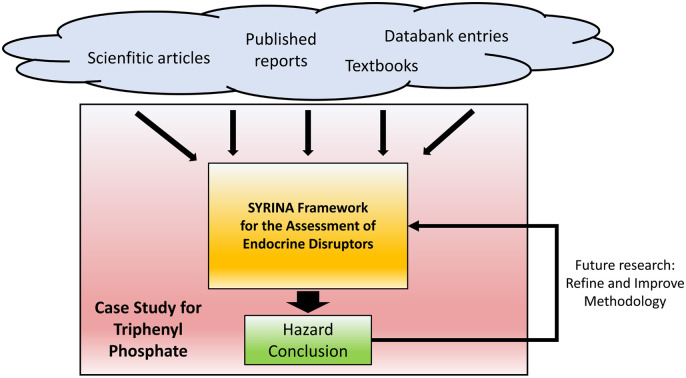

**Environmental significance**

Endocrine disruptors can have a high impact on the environment and since several decades, their importance was recognized within the scientific community and among regulators. This work provides insight into these chemicals and their effect from a molecular level to adverse outcomes in the whole organism. Further, it shows methods for summarizing, synthesizing, and evaluating available information in order to perform a hazard assessment for human health and the environment. Using a chemical with high amounts of data available, we show how our framework performs in terms of practicability, reproducibility, and scientific soundness. This methodology can then be further refined in order to improve the assessment of endocrine disruptors for both humans and non-target organisms in the environment.

## 1 Introduction

Systematic review is a methodology for evidence synthesis that uses pre-established, protocol-driven methods aimed at minimizing inconsistencies while increasing reproducibility and transparency of the results.^1,2^ Systematic reviews are already well-established tools in healthcare for assessing the efficacy of interventions or to investigate diagnostic tests or adverse outcomes.^3^ Systematic reviews have been recently adopted for use in chemical risk assessments (CRAs) as well^4–6^ and have been recommended by scientists and regulators.^7–11^ In the European Union (EU), systematic review approaches are also promoted in regulatory assessments of chemicals.^12^ It is important, however, to differentiate between CRAs that are conducted specifically for regulatory purposes, *e.g.* under EU or US legislation for the regulation of chemicals, and CRAs that are conducted by research teams/organizations and driven by a scientific interest or societal concern, because both types of CRA require different aspects to be considered and highlighted. For example, for regulatory purposes, only accepted criteria and methods, requiring years of implementation and validation, are important, whereas for scientific research, non-standard methods (studies, calculations, modelling) can also be utilized.

The advantages, challenges, and potential use of systematic reviews in this context have been previously discussed by Whaley *et al.*^13^ Examples of research needs highlighted by the authors include the development of methods for evaluating the internal validity (or “risk of bias”) of individual studies, defining a “gold standard” for conduct of systematic reviews and corresponding case-studies to explore how readily systematic review procedures can be integrated into the CRA process, especially for regulatory purposes.

Endocrine disruptors have received much attention from scientists and regulators for decades due to their potentially adverse effects on human health and the environment, as well as challenges in testing and identifying disruptions of the endocrine system.^14–16^ The importance of EDs and their impact on wildlife and humans has been highlighted in several reports published by the United Nations,^17,18^ most recently in the Global Chemicals Outlook II's emerging policy issues.^19^ In particular, exposure to EDs have been linked to several adverse health outcomes in humans such as decreased sperm quality,^20^ obesity and diabetes^21^ and breast cancer.^22,23^ In animal studies, effects such as thyroid hormone disruption^24^ or estrogenic and anti-androgenic effects^25,26^ have been observed. Moreover, EDs can have a significant effect on wildlife at the individual level^27,28^ as well as the population level.^29–33^

The World Health Organization (WHO) International Programme on Chemical Safety (IPCS) has provided a definition of an ED, which has been widely-accepted: “An endocrine disruptor is an exogenous substance or mixture that alters function(s) of the endocrine system and consequently causes adverse health effects in an intact organism, or its progeny, or (sub)populations”.^17^ This definition is used for regulatory purposes *e.g.* in the EU, and is, for example, the basis for the implemented criteria for identifying EDs within the regulations for plant protection products and biocidal products in the EU.^34,35^ The EU criteria state that for a chemical to be identified as an ED, it needs to show: (1) an adverse effect, (2) endocrine activity, and (3) that the observed adverse effect is causally linked to the endocrine activity. The criteria further state that available evidence should be evaluated using weight of evidence assessment, applying systematic review methodology to retrieve and summarize evidence from the open literature and other databases. The European Chemicals Agency (ECHA) and European Food Safety Authority (EFSA), supported by the European Commission's Joint Research Centre (JRC), have published a guidance document for the identification of EDs in the context of the Plant Protection Products (PPP) and Biocidal Products regulations.^36^

In 2016, an international research initiative including researchers, as well as experts from several international organizations, proposed a framework for “systematic review and integrated assessment (SYRINA)” for EDs (Vandenberg *et al.*^37^). While no regulatory criteria for the identification of EDs were yet in place, this initiative aimed to incorporate the principles of systematic review for identifying EDs in accordance with the WHO/IPCS definition. The SYRINA approach differs somewhat from the approach to identify EDs described in the ECHA/EFSA guidance,^36^ but the aspects of anchoring the identification in the WHO/IPCS definition and to implement systematic review methodology, especially for evidence retrieval, are similar. The aim of this study was to test the SYRINA framework by conducting a case-study evaluating the endocrine disrupting potential of triphenyl phosphate (TPP). TPP is widely used as a flame retardant, for example in the commercial mixture Firemaster 550.^38^ It is suspected to exhibit endocrine disrupting properties^39,40^ and is placed on the substance evaluation list (Community Rolling Action Plan, CoRAP) of the Registration, Evaluation and Authorization of Chemicals (REACH) regulation for potential endocrine disruption.^41^ Furthermore, it was determined that enough data exists for TPP to be a suitable chemical for this case study.

Our objectives were to evaluate the feasibility of applying SYRINA as a framework for assessing the ED potential of a relatively data-rich chemical substance and make recommendations for the further development of SYRINA based on the practical experience acquired when conducting the case study. In addition, we focused on an ED conclusion with relevance for human health. Assessment for other non-target (non-mammal) species were not part of the work, although they were presented for the sake of completeness. However, in an actual assessment they could be used as supporting information. We emphasize that in our case study, we were not attempting to come to a final conclusion about the ED status of TPP, but test the overall feasibility of SYRINA framework as a sequence of steps that could yield such a conclusion.

## 2 Materials and methods

**Fig. 1:**
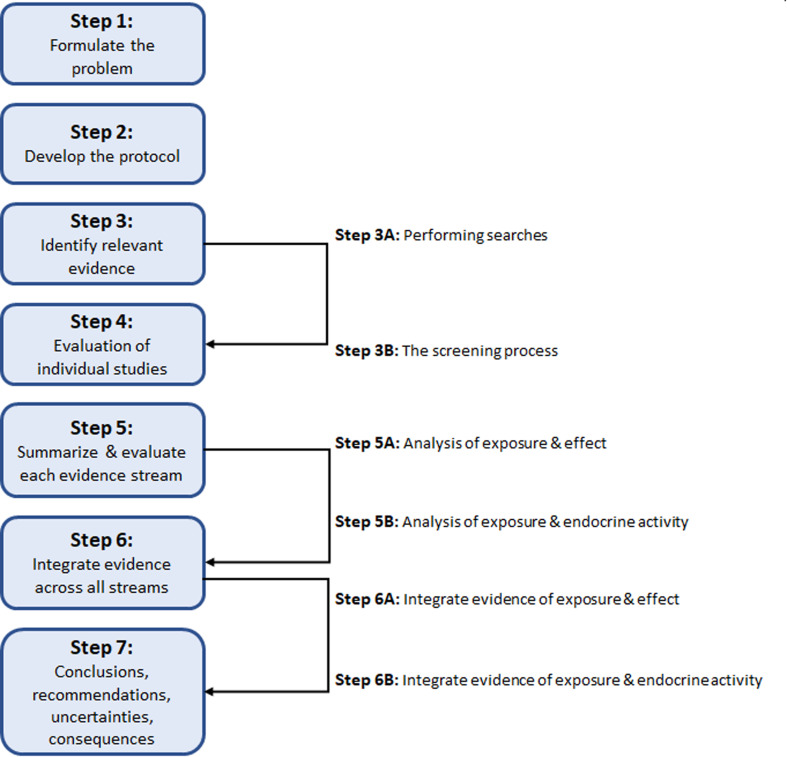
Overview of the of steps involved in the SYRINA framework according to Vandenberg *et al.* (2016).^37^

For this case study, we used the 7 steps of SYRINA (Fig. 1) as described in Vandenberg *et al.*,^37^ as the starting point. It was intended to follow SYRINA as close as possible, however, modifications we made and additional tools, which are not explicitly described in Vandenberg *et al.* (2016),^37^ are presented in Section 3. Whenever meaningful, a conclusion on the results obtained and the lessons learned for each step of SYRINA was provided.

Step 1 of SYRINA involves the interpretation of the research question (“what are the ED properties of X?”) as a Population Exposure Comparator Outcome statement. Step 2 is the development of a protocol, defining in advance of the conduct of a systematic review, the methods which will be used, including the search methods, eligibility criteria, study appraisal methods *etc.* Step 3 is the conduct of the literature search, where research and grey literature databases are used to retrieve evidence of potential relevance to the research question. The search results are then screened against the eligibility criteria for inclusion into the review. Step 4 is the critical appraisal of the included studies. Step 5 divides the evidence into streams (all epidemiological/wildlife, *in vivo* mammal, *in vivo* non-mammal, *in vitro* evidence for a particular outcome) and synthesizes the studies within each stream according to (a) evidence for adverse effects, and (b) evidence for endocrine activity. This represents the first two criteria of the WHO IPCS definition. Step 6 integrates the evidence streams for each criterion (a) and (b), rating the strength of evidence for each. Step 7 integrates (a) and (b), drawing of an overall conclusion to the original question of the ED identification of a chemical substance. These steps are summarized in Fig. 1.

### 2.1 Problem formulation – SYRINA Step 1

We followed the SYRINA framework by first formulating the problem in form of PECO statements where all modes of action, and routes and timings of exposure are included. This allowed testing SYRINA for large volumes of data, without restricting the outcome. This would be expected for current regulatory approaches to reviewing evidence of potential EDs. It is important to stress that all elements of the PECO statement are pre-defined depending on the research question.

### 2.2 Protocol development – SYRINA Step 2

Step 2 of SYRINA was not addressed as method development is a part of the case study. A typical systematic review would have a method available and agreed upon prior to conducting the main task, which is to address the ED potential of a particular chemical. In contrast, our focus lies upon the application, improvement and lessons learned from the SYRINA case study. It was not possible to develop one particular set of methods prior to performing the assessment. Instead, the methods (such as summarizing and integrating evidence streams) used in this work were a result of trial and error, modified during their application on the case study substance, as well as resulting from frequent discussions between the evaluating authors.

### 2.3 Identification of relevant evidence – SYRINA Step 3

#### 2.3.1 Literature search (Step 3a)

Literature searches, including identifying and applying the correct search terms, were performed using the databases EMBASE, Pubmed, SCOPUS, and Web of Knowledge. Alternative data sources (grey literature or other databases) were considered outside the scope of this work since it was assumed that unpublished study reports (*e.g.* by industry) cannot be obtained and therefore, not evaluated. As this substance has been the subject of regulatory reviews by ECHA and we are aware that industry-owned study reports could be reviewed by regulatory authorities, these are not part of this SR since these are not available to the authors. The consideration of such study results could have substantial influence on the overall conclusion on this specific substance. Since this work primarily focuses on methodology and not the substance itself, this was not regarded as a major problem. Furthermore, *in silico* data was considered outside the scope of this work because it would not add to the animal and mechanistic data for TPP significantly. Additionally, we think that it is more important to focus on the methodology to evaluate and assess experimental/epidemiological studies, in the context of an SR framework, as these are currently, especially from a regulatory view, usually considered the most relevant results. Although acceptance of *in silico* results has been increasing, the value of such methods is substantially lower than human/animal/*in vitro* data. No restrictions were placed on publication date or publication type, but language was limited to English. The exact search terms for each database can be viewed in the ESI (SI-1†). In general, they consisted of substance identification, effect identification and population identification. The search was performed with the help of a trained librarian in June 2018. For SCOPUS, only the first 2000 references (sorted by relevance) were included due to results display limitations of the database. Unfortunately, it was not possible to extend the display of references on SCOPUS at the time of search. This is an inherent function of the database and cannot be solved or otherwise modified by the user. As such, there was no way for the authors to see further than the first 2000 references. This is not believed to be of major concern as the search was performed on three additional databases.

#### 2.3.2 Literature screening (Step 3b)

Screening of references was successfully conducted in the software DistillerSR by three reviewers. No duplicate screening was performed, and assignment was done randomly. Although we found the program suitable for use in SYRINA, it should be noted that we did not fully test the capabilities of the program as it can also be used for other tasks within a systematic review (*e.g.* study evaluation, data extraction). After importing references, duplicates were removed using the software's function. Then, titles and abstracts were screened (Level 1) with references excluded according to the following exclusion criteria:

(1) Wrong chemical.

(2) No chemical tested.

(3) TPP not dosed as a single substance (*e.g.* in mixtures only).

(4) No exposure of chemical (*e.g.* synthesis of chemical).

(5) No testing of biological effects.

(6) Acute toxicity or unspecific mortality only.

(7) Not an original study that can be evaluated for risk of bias/internal validity (*e.g.* review articles, letters, short communications, *etc.*).

(8) Not written in English.

Following title/abstract screening, full text was screened (Level 2) by one reviewer, applying the same exclusion criteria as listed above.

### 2.4 Evaluation of individual studies – SYRINA Step 4

#### 2.4.1 *In vitro* and *in vivo* toxicity studies

Several study evaluation tools are described in Vandenberg *et al.*^37^ For this case study, we used the Science in Risk Assessment and Policy (SciRAP) tool (Beronius *et al.* (2018)^42^; Roth *et al.* (2021)^43^) for the evaluation of *in vitro* and *in vivo* toxicity studies. The SciRAP tool was developed to evaluate reliability and relevance of toxicity studies for use in hazard and risk assessment of chemicals using specific pre-defined criteria and is freely available at www.scirap.org. In order to modify the tool for use in systematic reviews, we applied modifications by first including additional criteria for the study evaluation, in particular for blinding and attrition (see Table SI-1†). Evaluation of relevance was not conducted here since this is covered by the PECO statements and the study screening process.

Our modified SciRAP method therefore assesses (1) reporting quality and (2) methodological quality (including internal validity and study sensitivity) of each individual study. Evaluations were performed by two independent reviewers (one main and one supporting evaluator) on the Health Assessment Workspace Collaborative (HAWC) platform (a database used by the US EPA to conduct systematic reviews).^44^ For each criterion, the following ratings were given: fulfilled, partially fulfilled and not fulfilled. Then, as a final outcome, a “final confidence rating”, representing the overall confidence in the methods and results of the study, is given. The final confidence ratings used were high, medium, low, or uninformative. No explicit cut-off criteria (*e.g.* percentage or threshold value) were applied and this judgement is based purely on expert opinion, for example considering the amount of fulfilled, partially fulfilled and not fulfilled criteria as well as how important the criteria were for the overall confidence of the study. This was determined by the main reviewer (one of the two study evaluators) after careful consideration of ratings and details from both reviewers. Uninformative studies were eliminated from further study evaluation.

At the time of evaluation, the SciRAP tool for evaluating *in vitro* studies was still undergoing review and development. However, no other study quality method for *in vitro* data was readily available and thus the preliminary version of the SciRAP tool was used for this work. In other systematic reviews used to assess chemical hazard, *in vitro* mechanistic data has only been used as supporting information and the studies were not evaluated individually.^45,46^ Here, we explored the possibility of treating mechanistic data and animal toxicity data equally and therefore applied the modified SciRAP evaluation tool also on individual *in vitro* results.

#### 2.4.2 Epidemiological studies

The SciRAP platform does not include a tool for evaluation of epidemiological studies, and we recognize the major challenge to be the difficulty in finding study evaluation tools with the same construct to use for all types of studies, namely *in vivo*, *in vitro*, and epidemiological studies. We therefore used the IRIS tool for evaluating the epidemiological studies in this work. Since it did not give detailed criteria, the evaluation required expertise and guidance. However, a document, containing core and prompting questions and instructions, was available on the HAWC platform. This tool assesses risk of bias but also addresses study sensitivity. The IRIS tool was not modified in any way for the evaluation of epidemiological studies. As for the *in vivo* and *in vitro* studies, evaluations were done on the HAWC platform. See Table SI-2† for the criteria used. Again, four ratings for the overall confidence were used and uninformative studies were omitted from further consideration as described above.

### 2.5 Summarize and evaluate each evidence stream – SYRINA Step 5

Step 5 of the SYRINA framework consists of evaluating the strength of each stream of evidence. For this, (a) the association between exposure and adverse effect for *in vivo* and epidemiological studies and (b) the association between exposure and endocrine activity for *in vivo* and *in vitro* studies were assessed. A stream of evidence is defined as all epidemiological or *in vivo* or *in vitro* data for all outcome groups. We use the term “outcome group” to differentiate between types of outcomes, for example neurodevelopment or cardiovascular effects. As a consequence, streams of evidence represent the first level of grouping into human, wildlife, *in vivo* mammals, *in vivo* non-mammals, and *in vitro* evidence. As a second level, these streams have been divided into outcome groups.

All measured endpoints are assigned to a particular outcome group and therefore, each outcome group will have one or several streams of evidence, which can be related to evidence of adverse effect or evidence of endocrine activity. It is therefore entirely possible that several streams of evidence for different endocrine modes of action are linked to the same (or different) outcome groups.

#### 2.5.1 Data extraction

Data were extracted for all evaluated studies except for the seven uninformative studies mentioned above (see Table SI-3†). This step was carried out on the HAWC platform which provides data extraction templates for epidemiological, *in vivo*, as well as *in vitro* studies. Where needed, an online tool (https://automeris.io/WebPlotDigitizer/) was used to extract numerical data from figures and graphs. Extracted data as well as all included studies can be viewed in detail on the HAWC website of the project (https://hawcprd.epa.gov/assessment/100000054/).

#### 2.5.2 Generating outcome groups

The extracted data were exported to a Microsoft Excel spreadsheet and all extracted endpoints were grouped together according to the type of evidence, forming an outcome group. This step was performed by three assessors, including discussion and expert opinion.

#### 2.5.3 Evaluating outcome groups

**Fig. 2:**
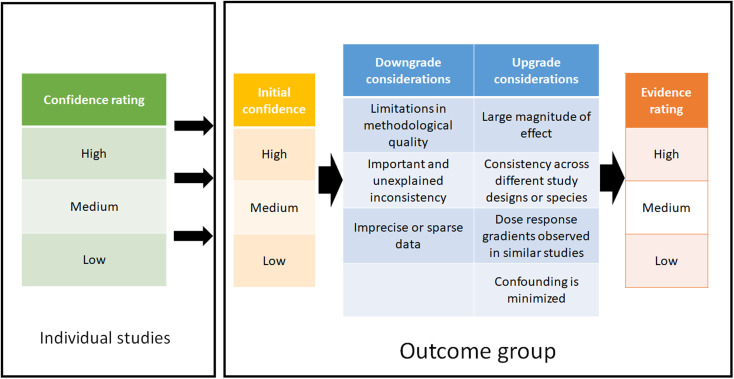
Determination and summary of confidence rating for each included study and evidence ratings for each outcome group.

To determine the strength of evidence of an outcome group, an approach using aspects of the GRADE method^47^ was used. The outcome group, consisting of one or several studies and endpoints, can be upgraded or downgraded according to the considerations shown in Fig. 2. At first, the outcome group was assigned an initial confidence rating, depending on the study type. Outcome groups formed by *in vivo* and *in vitro* studies were assigned a “high” initial confidence rating, whereas those formed from epidemiological studies were assigned a “medium” initial confidence rating.

Expert judgment was utilized when down/upgrading the evidence and when determining a final rating for each outcome group. This was performed by three reviewers, where an agreement had to be reached between all evaluators. It should be emphasized that the confidence ratings for the individual studies have no influence on the initial confidence rating of the outcome group. However, confidence ratings of the studies are taken into consideration *e.g.* when downgrading the evidence due to “limitation in methodological quality”. Both methodological and reporting quality play a role in determining this downgrade consideration, *i.e.* if these qualities were deemed low, this would lead to a downgrade of the overall evidence rating.

### 2.6 Evidence integration across all streams – SYRINA Step 6

Step 6 of the SYRINA framework involves integrating the separate evidence streams to come to overall conclusions about (a) the strength of evidence for an adverse effect, and (b) the strength of evidence for endocrine activity. SYRINA proposes a matrix-based approach for making this determination.^37^ First, the final evidence ratings for each outcome group were grouped together, for example, evidence from human/wildlife were paired with experimental *in vivo* data for one specific outcome group. Here, special considerations were taken whether the data from one outcome group showed an effect or not. For example, even though the evidence was rated medium after down- or upgrading, if they pointed to the absence of any effect in this outcome group, the final evidence rating will be set to “no evidence of effect” instead of “medium”. For Step 6a, the final evidence ratings based on human or wildlife observational data were compared to experimental *in vivo* data for the same outcome group. This allowed for the determination of the strength of the evidence for the association between exposures and adverse effect. Similarly, for Step 6b the same procedure was applied, comparing *in vitro* and experimental *in vivo* data for endocrine activity.

### 2.7 Conclusions, recommendations, uncertainties, consequences – SYRINA Step 7

According to the SYRINA framework, the final step combines the evidence in Step 6 using a final matrix (Fig. 5 in Vandenberg^37^). This also involves assessing the third criterion of the ED definition, that is the causal link between adverse effects and endocrine activity.

## 3 Results of applying the TPP SYRINA review methodology

### 3.1 Problem formulation – SYRINA Step 1

The Populations, Exposures, Comparators and Outcomes (PECO) statement for TPP can be seen in Table 1. The PECO statement anticipates five streams of evidence (human, wildlife, *in vivo* mammal, *in vivo* non-mammal and *in vitro*) and is inclusive of all modes of action, and routes and timings of exposure. As mentioned in Section 2.1, all elements of the PECO statement are pre-defined depending on the research question. For example, the outcomes presented in Table 2 aim to cover all possible endocrine related endpoints and do not depend on properties of information available for TPP.

**Table 1:** Population, Exposure, Comparator and Outcome (PECO) statements for the SYRINA case study on TPP

Population	Exposure	Comparator	Outcomes
*In vitro* systems: animal/human cell lines or tissue models	Triphenyl phosphate (TPP; TPHP; CAS# 115-86-6)	*In vivo* and *in vitro*: exposed groups *vs.* negative/vehicle controls, positive control if available	Endocrine related endpoints, including thyroid system, sex hormones, neuroendocrine system, renin–angiotensin system (RAS) and energy homeostasis
Animals (*in vivo*, any developmental stage)	No restrictions on timing or route of exposure	Epidemiology: high exposure *vs.* low exposure groups	Developmental-, reproductive-, neuro- and immunotoxicity
Human (epidemiology, occupational and general population)			Teratogenicity, effects on metabolism, carcinogenicity

**Table 2:** Outcome groups and streams of evidence for different study types following data extraction

Epidemiological	*In vivo* mammal	*In vivo* non-mammal	*In vitro*
Male reproductive system	Male reproductive system	Male reproductive system	Estrogen activity
Neurodevelopment	Female reproductive system	Female reproductive system	Androgen activity
Immune system	Reproductive function	Reproductive function	Steroidogenesis
	Neurodevelopment	Neurodevelopment	Mineralocorticoid and glucocorticoid activity
	Metabolism	Neurotoxicity	Thyroid system
	Immune system	Metabolism	Lipid metabolism
	Development & growth	Cardiovascular system	Glucose homeostasis
	Androgen activity	Adult growth	Cardiovascular system
	Thyroid system	Development & growth	Hypothalamic–pituitary–adrenal (HPA) axis
	Lipid metabolism	Estrogen activity	Renin–angiotensin–aldosterone system (RAAS)
	Glucose homeostasis	Androgen activity	Aryl hydrocarbon receptor (AhR) activity
	Growth hormones	Steroidogenesis	Peroxisome proliferator-activated receptors (PPAR) activity
	Cardiovascular system	Thyroid system	Retinoic acid receptor/retinoic X receptor (RAR/RXR) activity
		Lipid metabolism	
		Glucose homeostasis	

### 3.2 Protocol development – SYRINA Step 2

As described in Section 2.2, Step 2 of SYRINA was not addressed in this case study.

### 3.3 Identification of relevant evidence – SYRINA Step 3

**Fig. 3:**
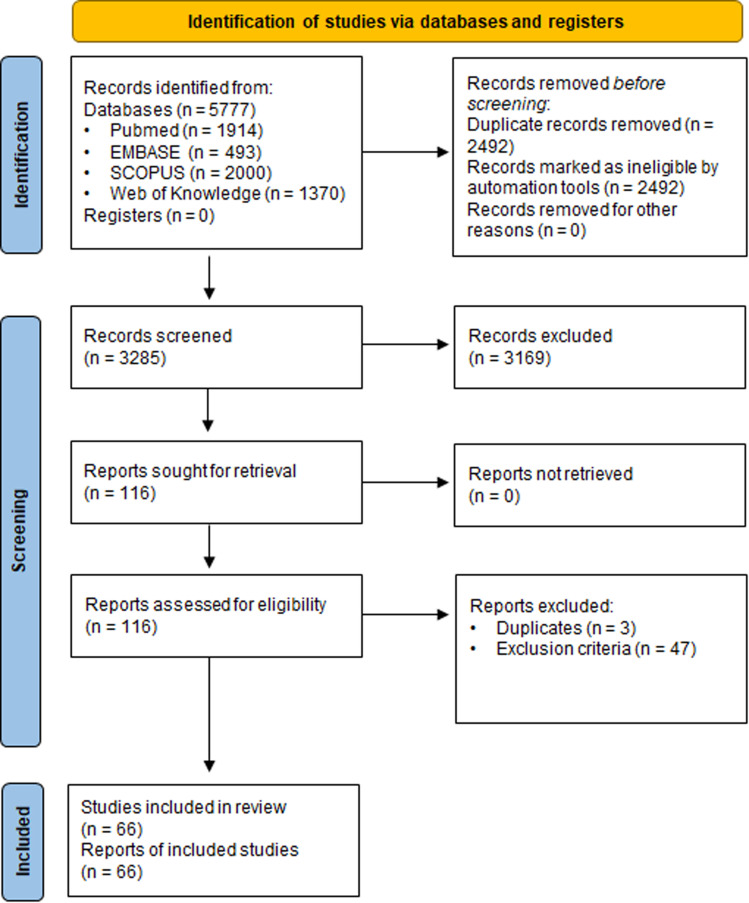
PRISMA flow diagram for the literature search and screening procedure as well as number of references at each step. Records refer to the abstract/title screening step whereas reports refer to the full text screening step.

The literature search resulted in 5777 references; these were imported (Fig. 3). After removal of duplicates, 3285 underwent title/abstract screening, leaving 116 studies for full text screening. At the end of the screening process, 66 studies (6 epidemiological studies, 19 animal studies and 42 *in vitro* mechanistic studies with one study being counted in both *in vivo* and *in vitro* categories) were included for further evaluation.

### 3.4 Evaluation of individual studies – SYRINA Step 4

In total, we rated 19 studies as having high confidence, 25 as having medium confidence, 16 as having low confidence and seven as being uninformative. Uninformative studies were not further considered.

#### 3.4.1 *In vivo* studies

**Fig. 4:**
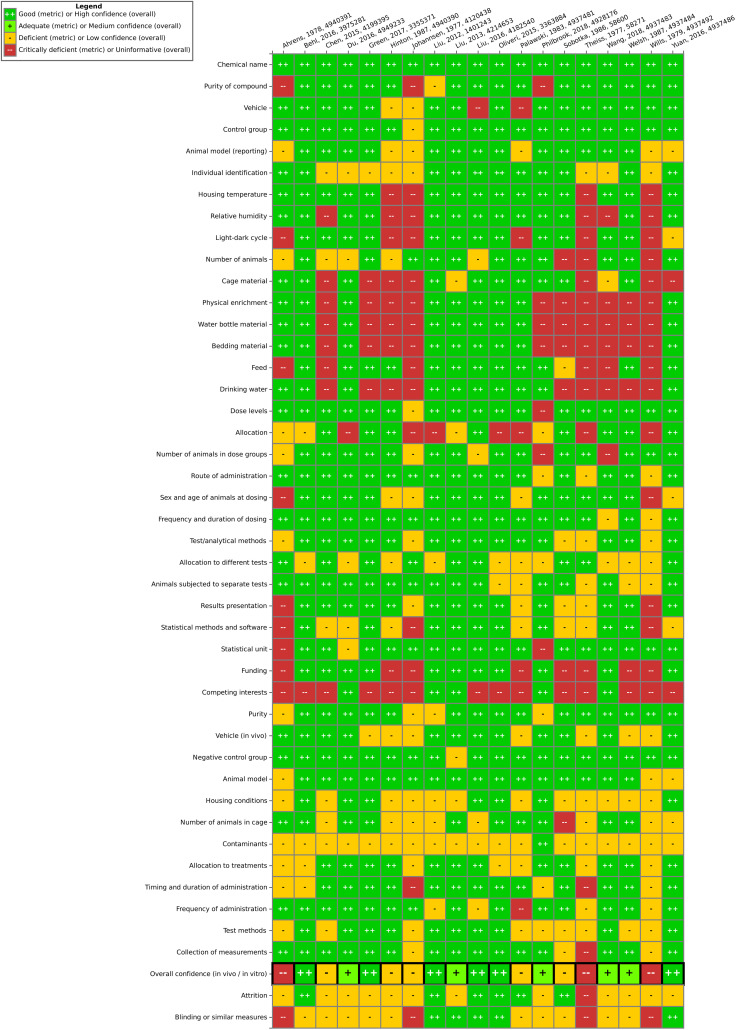
Study evaluation results for *in vivo* studies using the SciRAP tool. “Good” corresponds to “fulfilled”, “deficient” corresponds to “partially fulfilled” and “critically deficient” corresponds to “not fulfilled”. Expert judgment was utilized to arrive at the overall confidence, taking into consideration the number of certain ratings as well as the importance of a specific criterion. This figure can also be found at https://hawcprd.epa.gov/summary/visual/100500019/.

Results for *in vivo* data (Fig. 4) showed six studies with high confidence, five with medium confidence, five with low confidence and three studies being uninformative. It could be observed that for reporting quality, information related to housing conditions (*e.g.* physical enrichment, water bottle material, bedding material, and drinking water) as well as funding and competing interests were often insufficiently reported. Consequently, criteria concerning housing conditions and contamination issues in the methodological quality section were often only partially fulfilled (seen as “deficient”). Blinding and attrition were also rarely fulfilled. Generally, exposure conditions, animal model information and controls were well reported and suitable. The three uninformative studies (see Table SI-3†) had generally poor reporting and/or other serious flaws and were excluded from further analysis.

#### 3.4.2 *In vitro* studies

**Fig. 5:**
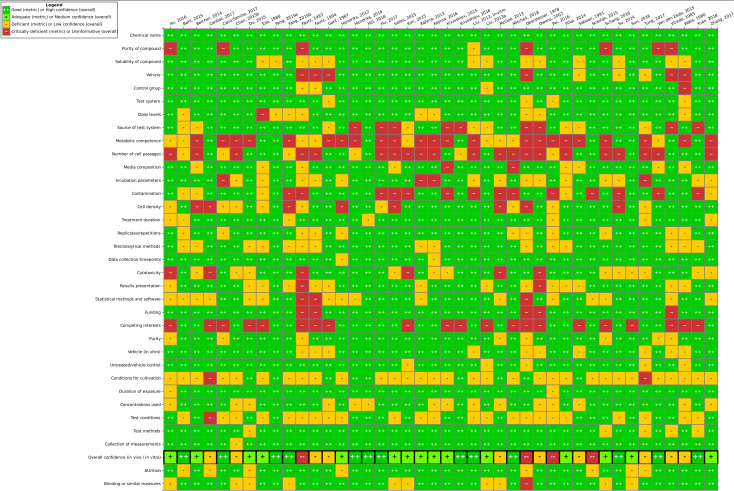
Study evaluation results for *in vitro* studies using the SciRAP tool. “Good” corresponds to “fulfilled”, “deficient” corresponds to “partially fulfilled” and “critically deficient” corresponds to “not fulfilled” in SciRAP. Expert judgment was utilized to arrive at the overall confidence, taking into consideration the number of certain ratings as well as the importance of a specific criterion. This figure can also be found at https://hawcprd.epa.gov/summary/visual/100500018/.

For *in vitro* data, 13 studies were rated as high confidence, 15 were rated as medium confidence, 10 were rated as low confidence and four were deemed uninformative (Fig. 5). It was often seen that the source of test system, metabolic competence and number of cell passages were not well reported. Competing interest was, similarly to the *in vivo* studies, frequently not reported. The methodological quality of most studies was relatively high and “partially fulfilled” criteria were often observed due to issues with cultivation and test conditions. The four excluded uninformative studies (Table SI-3†) were in two instances due to reporting/methodological flaws and in two instances due to being irrelevant to the PECO statement (only acute toxicity or cytotoxicity measured).

#### 3.4.3 Epidemiological studies

**Fig. 6:**
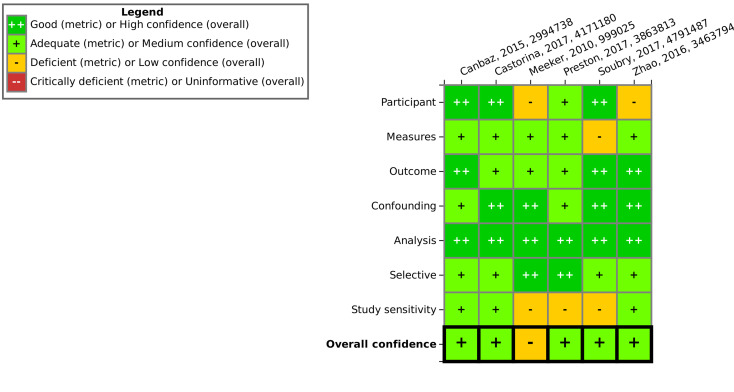
Study evaluation results for epidemiological studies using the IRIS tool. Expert judgment was utilized to arrive at the overall confidence, taking into consideration the number of certain ratings as well as the importance of a specific criterion. This figure can also be found at https://hawcprd.epa.gov/summary/visual/100500039/.

The results of the evaluation of epidemiological studies are shown in Fig. 6. Of the six evaluated epidemiological studies, five were found to be of medium confidence while for one study, low confidence was concluded due to flaws in participant selection and study sensitivity.

### 3.5 Summarize and evaluate each evidence stream – SYRINA Step 5

An overview of the established outcome groups mentioned in Section 2.5 is given in Table 2, whereas a more detailed presentation of the grouping of endpoints according to effect type and study type, including the distinction between adverse effects and endocrine activity, can be seen in the SI-2.† Study details, summaries of results, upgrade/downgrade considerations and the final ratings for the outcome groups and reasoning for the conclusions are shown in tabular form in the ESI.†

#### 3.5.1 Human, wildlife and experimental *in vivo* data relevant for the assessment of adverse effects

Table 3 shows the evidence ratings for adverse effects for the different outcome groups “human” as well as “experimental *in vivo* data for mammals”. Where no data for an outcome group was found for human/wildlife or *in vivo* data, this was marked as “no data”. Data not showing significant effects were marked “no evidence for effect” with its final confidence rating from the previous step after down-/upgrading considerations in brackets. It should be noted that “no evidence for effect” should only be seen in the context of this assessment as proving a negative in general is not possible.

**Table 3:** Evidence ratings for outcome groups determined for adverse effects in humans/wildlife and experimental *in vivo* studies

Outcome group	Human (mammal)	Experimental *in vivo* (mammal)
Thyroid system	Low	No data
Cardiovascular system	Low	No data
Male reproductive system	Low	Low
Female reproductive system	No data	No evidence for effect (medium)
Reproductive function	No data	Medium
Neurodevelopment	Low	No evidence for effect (low)
Immune system	No evidence for effect (low)	No evidence for effect (medium)
Glucose homeostasis	No data	No data
Metabolism	No data	Medium
Development & growth	No data	No evidence for effect (medium)

Table 4 shows the evidence ratings for wildlife as well as experimental *in vivo* data for non-mammals. As with the mammal-data above, no data for an outcome group and data not showing significant effects were also marked “no data” and “no evidence for effect” respectively. It could be observed that for non-mammals, no wildlife information for any outcome group was available, hence the body of evidence was only consisting of experimental *in vivo* data. Non-mammalian data is presented for the sake of completeness only. Further evidence synthesis was therefore only performed for mammalian data.

**Table 4:** Evidence ratings for outcome groups determined for adverse effects in non-mammal wildlife and experimental *in vivo* studies

Outcome group	Wildlife (non-mammal)	Experimental *in vivo* (non-mammal)
Male reproductive system	No data	No evidence for effect (medium)
Female reproductive system	No data	Medium
Reproductive function	No data	Medium
Neurodevelopment	No data	High
Metabolism	No data	No evidence for effect (low)
Cardiovascular system	No data	High
Development & growth	No data	High
Growth (adults)	No data	Low

#### 3.5.2 Experimental *in vivo* and *in vitro* data relevant for the assessment of endocrine activity

Similar to the assessment of adversity, the results for the evidence ratings for endocrine activity of outcome groups are shown in Table 5. Experimental *in vitro* based on non-mammalian and mammalian cells were summarized together, as it was assumed that the endocrine systems are similar enough to draw conclusions about the mechanistic effects of the endocrine system, regardless of species. However, *in vivo* results were still treated separately and only the mammalian results were used in further assessments. The outcome groups “AhR activity”, “PPAR activity”, “MC and GC activity”, and “RAR/RXR activity” were treated as ESI† for other outcome groups, as these can potentially have an effect on multiple endocrine systems. For the lipid metabolism, the experimental *in vitro* data were regarded as “high” because of clear effects on PPAR activity.

**Table 5:** Evidence ratings for outcome groups determined for endocrine activity in mammals and non-mammals and *in vitro*

	Experimental *in vivo* mammals	Experimental *in vivo* non-mammals	Experimental *in vitro*
Anti-androgen activity	Low	Medium	High
Estrogen activity	No data	High	High
Steroidogenesis	No data	High	High
Thyroid system	No evidence for effect (medium)	No evidence for effect (medium)	Medium
Lipid metabolism	No data	Medium	High (aPPARg)
Growth hormone/IGF-1 (adult)	Medium	No data	No data
Growth hormone/IGF-1 (developmental)	Medium	No data	No data
Immune system (developmental)	Low	No data	No data
Glucose homeostasis	No data	No data	Medium
AhR activity	No data	No data	No evidence for effect (low)
PPAR activity	No data	No data	High
Mineralocorticoid and glucocorticoid activity	No data	No data	High
RAR/RXR activity	No data	No data	Medium

a High rating due to effects observed on peroxisome proliferator-activated receptor gamma as supporting information.

### 3.6 Evidence integration across all streams – SYRINA Step 6

The most solid evidence for adverse effects was shown for the outcome groups “reproductive function” as well as “metabolism” (Table 3, with supporting information in Table 4). Therefore, the integration of evidence for drawing a conclusion on ED potential was focused on these two outcome groups. With regards to ED activity, evidence integration focused on effects on estrogen and anti-androgen activity, steroidogenesis, lipid metabolism, growth hormone/IGF-1 for (adult and developmental) and glucose homeostasis.

#### 3.6.1 Human, wildlife and experimental *in vivo* effects

**Fig. 7:**
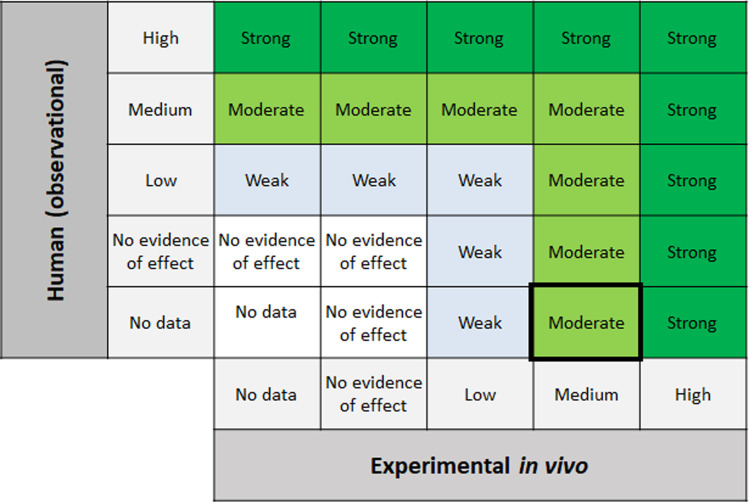
Adverse effects evidence integration for outcome groups “reproductive function” and “metabolism”.

Using the SYRINA matrix, evidence ratings for adverse effect groups are shown in Fig. 7. For both “reproductive function” and “metabolism”, the absence of observational data and medium evidence for adverse effects lead to a final strength of “moderate” for the evidence (marked with a black box).

#### 3.6.2 Endocrine activity

**Fig. 8:**
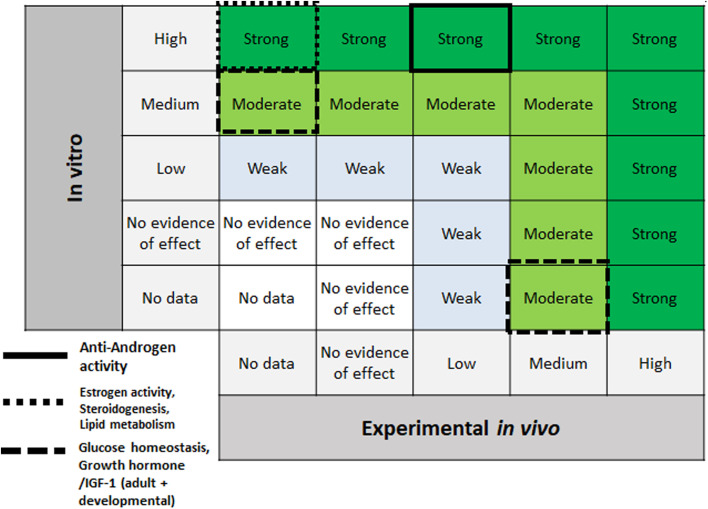
Endocrine effects evidence integration for outcome groups “anti-androgen activity” (solid line), “estrogen activity” + “steroidogenesis” + “lipid metabolism” (dotted line), and “glucose homeostasis” + growth hormone/IGF-1 (adult + developmental) (dashed line).

Evidence ratings for endocrine effect groups are shown in Fig. 8, marked with different black boxes. For anti-androgen activity, high confidence from *in vitro* and low confidence from *in vivo* mammalian data lead to the final rating of “strong” for the evidence. For estrogen activity and steroidogenesis, a final rating of “strong” was achieved by combining high confidence *in vitro* data with the lack of *in vivo* mammalian data. Lipid metabolism showed strong evidence due to high confidence from *in vitro* data and strong supporting evidence from PPAR activity, as well as “no data” for *in vivo* studies. Moderate strength of evidence for glucose homeostasis was determined as no *in vivo* data were present, and *in vitro* evidence was rated “medium”. Lastly, moderate strength of evidence for growth hormone/IGF-1 (adult + developmental) was determined due to no data present *in vivo*, and “medium” confidence *in vitro*.

### 3.7 Conclusions, recommendations, uncertainties, and consequences – SYRINA Step 7

A critical aspect of the ED assessment is to conclude on biologically plausible links between identified endocrine activities and observed adverse effects. For this case study, we performed a non-systematic analysis of the links between observed adverse effects and endocrine activities. This was done for the two strongest lines of evidence for endocrine disruption based on the previous step of SYRINA, which are (1) reproductive function (related endocrine activity outcome groups were: estrogenic and anti-androgenic activity as well as steroidogenesis) and (2) metabolic disruption. Due to the findings further described below, we found it necessary to include data on both lipid metabolism as well as glucose homeostasis and combine these into the one common outcome “metabolic disruption”.

Then, using a matrix approach described in Vandenberg *et al.*,^37^ a final conclusion on the ED identification was drawn with focus on human health. Effects and endocrine activity observed *in vivo* in non-mammals were only regarded as supporting evidence if inconclusive results were present in mammalian data.

#### 3.7.1 Establishing links between adverse effect and endocrine activity of TPP

##### Reproductive function

The review concludes that in terms of adverse effects, TPP may cause effects on some reproductive function parameters, primarily placental weight, although this was only observed in one study.^48^ Effects on placental weight is considered “sensitive to but not diagnostic of” interference with estrogen, androgen, thyroid, steroidogenesis or retinoid modalities.^49^ Other observed non-significant effects include decreases of litter sizes and increase of the percent resorptions in litter in mice^48^ as well as a decrease of males per litter,^50^ implantation efficiency, fetus viability, and implants per female.^51^ Lastly, a non-significant increase of male/female ratio in the litter could be observed.^51^

TPP was also shown to have estrogenic and anti-androgenic activity, and effects on steroidogenesis, *in vitro*. Non-mammalian results showed mostly changes in gene expression associated with anti-androgen^52^ or estrogen^53^ activity. In mammals, more evidence exists for estrogen activity,^54–57^ anti-androgen activity,^55,56,58^ and effects on steroidogenesis.^54,58–61^ No *in vivo* mammalian data are available for estrogenic activity. However, anti-androgenic activity could be observed in male mice due to decrease of testicular testosterone concentration combined with a decrease in gene expression of various genes responsible for testicular testosterone transport^62^ as well as increased hormone (among others, estradiol and prolactin) concentrations in human males.^63^ Furthermore, significant PPAR-gamma activity was observed in several studies,^38,64–66^ including one study using chinese hamster ovary cells.^67^

PPAR-gamma and its downstream targets were found to play a major role in regulating follicular rupture and ovulation in mice.^68^ In another study, the authors showed that treated pregnant wild-type mice with the PPARgamma agonist rosiglitazone resulted in a disorganization of the placental layers and an altered placental microvasculature, accompanied by the decreased expression of some proangiogenic genes and vascular endothelial growth.^69^ However, without further data it is not possible to describe this particular endocrine mode of action for the effects on reproductive function in detail. Nevertheless, we can overall conclude that there is sufficient evidence for a plausible link between adverse effects shown above and the endocrine activity observed in related *in vivo* and *in vitro* data.

##### Metabolic disruption

This review also concludes that TPP may have an adverse effect on metabolic parameters. Specifically, an increase of fat deposits alongside an increase of T2 diabetes incidences in mice were considered to be the adverse effects regarding metabolism.^50^

On the endocrine activity side, increased lipid accumulation,^38,65^ increased cholesterol and triglycerides, increased adipogenic differentiation in cells^66^ as well as effects on glucose homeostasis, including increased insulin resistance, increased glucose levels and increased glucose uptake have been reported for TPP.^48,50,66,70,71^

Obesity is commonly a major risk factor for T2 diabetes^72^ and lipid accumulation and/or increased adipogenic differentiation in cells can lead to increased fat deposits, as shown in Green *et al.*^50^ and Cano-Sancho *et al.*^66^ A high triglyceride level could be a risk factor for T2 diabetes as well^73,74^ and there is a strong correlation between increased plasma free fatty acids, leptin levels, lipid accumulation and insulin resistance.^75–79^ Also, large fat cells and the resulting increased plasma non-esterified fatty acid concentration are risk factors for the development of non-insulin-dependent diabetes mellitus.^80^ TPP was also shown in several studies to be a PPAR-gamma-agonist. PPAR-gamma is involved in processes for fatty acid storage, glucose metabolism, lipid uptake and adipogenesis.^81–83^ However, PPAR-gamma activation generally results in increased insulin sensitivity,^81,84,85^ which is contradictory to the results seen for TPP (increased insulin resistance). These mechanisms are however complex, and it is likely that many different mechanisms interact to cause the effects on metabolism observed for TPP. Lastly, according to a proposed adverse outcome pathway currently under development, interaction with PPAR-alpha could cause metabolic disruption, which indirectly supports the conclusions drawn in this study for PPAR-gamma.^86^ However, this information should be regarded with care as the pathway has not been established yet.

Overall, we see a well described link between the adverse effect (development of T2 diabetes) and the metabolic disruptions observed *in vivo* and *in vitro* regarding lipid and glucose metabolism.

#### 3.7.2 Identification of TPP as an ED

**Fig. 9:**
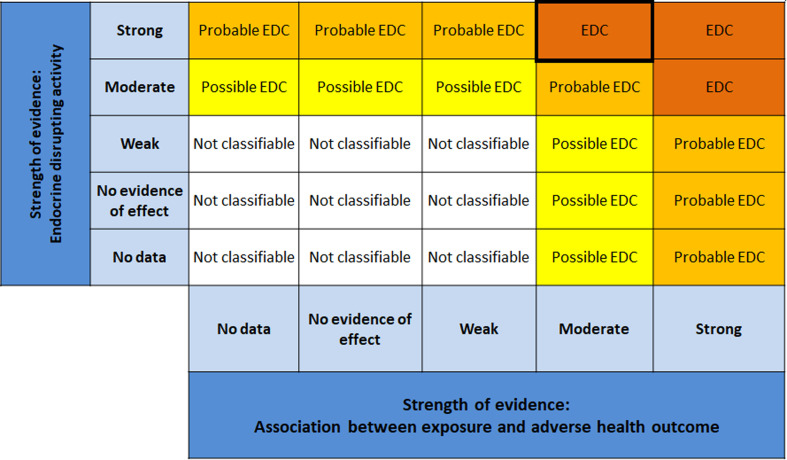
Matrix for drawing conclusions about endocrine disruption. EDC = endocrine disrupting chemical modified from Vandenberg *et al.* by including “no evidence of effect”.

We integrated evidence bodies from the assessment of adverse effects with corresponding evidence bodies from the endocrine activity assessment. The results can be observed in Fig. 9, where for both reproductive function and metabolic disruption, a final identification of “EDC” (marked with a black box) as a precautionary approach based on strong evidence for ED activity (Fig. 7) and moderate strength for adverse outcomes (Fig. 6). Overall, the final conclusion is based on the following information:

1. Reproductive function.

• 12 studied endpoints *in vivo* (3 unique studies), only mammalian data considered.

• 26 studied endpoints *in vitro* (8 unique studies), mammalian and non-mammalian data considered.

2. Metabolic disruption.

• 19 studied endpoints *in vivo* (5 unique studies), only mammalian data considered.

• 33 studied endpoints *in vitro* (12 unique studies), mammalian and non-mammalian data considered.

It is important to mention that several measured endpoints could originate from the same study or publication.

## 4 Discussion

### 4.1 Problem formulation – SYRINA Step 1

According to the PECO statements, no restrictions on the type of endocrine disruption nor on the relevant population (=species) were made. Because of the vast amounts of data available for TPP, this approach should be reconsidered in future applications of SYRINA. It is, however, important to consider the time requirements associated with the evaluation of such a large amount of information. If applying this approach in a regulatory context, and to make tasks more manageable, the evidence could be broken down into one mode of action at a time at first, although this concerns primarily later Steps 5–7 of the framework.

### 4.2 Protocol development – SYRINA Step 2

As this step was omitted (see reasoning above), no further discussion was performed here.

### 4.3 Identification of relevant evidence – SYRINA Step 3

We did not observe any major shortcomings of the methodology used in Section 2.3 in order to identify the relevant evidence. Hence, the same or similar approaches could be used in future applications of SYRINA. It should be noted that no search of grey literature or other databases was done. This highlights the importance of public availability of full study reports for independent evaluation and to enable a comprehensive assessment. The evaluation can be seen as partly incomplete due to the lack of access to unpublished studies. However, this evaluation could be used to complement a comprehensive assessment of the ED properties of TPP. Also, *in silico* approaches were not considered. Including these aspects could potentially lead to a high amount of information (*e.g.* industry studies) for Steps 4–7 and such inclusions would increase the workload substantially. However, *in silico* results would likely not have made significant impact on the results of this work since TPP already showed a large availability of *in vitro* and *in vivo* data in the literature.

### 4.4 Evaluation of individual studies – SYRINA Step 4

Overall, one finding of this work is that the steps of study evaluation tools such as SciRAP and IRIS's HAWC (Health Assessment Workspace Collaborative) could be followed in the context of the SYRINA framework, and that the results were usable as input for the next stage of the assessment.

It could be seen that the few epidemiological studies as well as most of the *in vitro* studies were found to be of good quality as only one deficient (out of 6 studies) and 10 deficient/4 critically deficient studies (out of 42 studies) were identified, respectively. *In vivo* animal studies were found more to be lacking in quality as out of 19 studies, almost half were found to be either deficient (5 studies) or critically deficient (3 studies). The results showed that for some types of studies, certain reporting or methodological quality criteria were often insufficiently fulfilled. For example, for *in vivo* studies, these were criteria concerning housing conditions and for *in vitro* studies, these were conditions for cultivation and test conditions. Funding and competing interests were also often insufficiently reported. As such, there is a need for improving the reporting as well as the methodological quality with respect to these specific criteria in literature studies. On the other hand, for the overall confidence of a study to be deemed “deficient” or worse, other, more important criteria were found to be decisive (and not fulfilled) such as purity of the compound, sex and age of the animals, timing and duration of dosing, contamination (*in vitro*), or concentrations used, among others. Although some studies were later removed for irrelevance, it is important to note that irrelevance according to the PECO statement should have been detected at the screening level and therefore these studies underwent evaluation due to oversight/screening error. Generally, we found that more diverse expertise was required due to the large number of diverse endpoints, test systems or types of tests presented. Similar to the *in vivo* SciRAP tool, the *in vitro* tool was relatively easy to use, and a final confidence rating reached by expert judgment was found to be the currently most suitable approach. A more detailed validation of this tool and a thorough analysis for use in systematic reviews would be beneficial for future applications of the framework. Lastly, we did not try to resolve differences in evaluations by discussion or by another independent party, as is typically done in other systematic review tools. Therefore, study evaluation results should be regarded with care and the overall conclusion on the ED properties of TPP should not be taken as final unless the proper steps are performed, including the development of a protocol. As a case study and a method testing work, we believe that these flaws do not significantly impact the main objective of this paper. Further improvements of this step for regulatory assessment could be achieved by introducing clearer criteria regarding the weighting of parameters leading to high or low overall confidence of a study to reduce the currently high expert judgment required. This includes defining criteria which could lead to an immediate rating of “critically deficient” if not fulfilled, for example for highly essential information such as the identity of the tested substance.

### 4.5 Summarize and evaluate each evidence stream – SYRINA Step 5

Using Step 5 of SYRINA, we have provided an evidence rating for each outcome group formed, differentiating between epidemiological, *in vivo* and *in vitro* data as well as between mammal and non-mammal data.

It is important to mention that the approach used to upgrade or downgrade the evidence could lead to an overrating (from an expert judgment point of view) of the evidence for some outcome groups. For example, when the data was found to be imprecise or sparse, a high initial confidence rating based on animal studies was downgraded only once, leading to medium evidence. One possible solution is to set criteria for downgrading twice if the downgrade consideration is strongly met. Alternatively, the initial confidence rating of an outcome group, based on the confidence ratings of the individual studies and not on the type of study performed, could be considered. As such, a revisiting of these possibilities given in GRADE is recommended. Another observation made was the high amount of gene expression data available. In many cases, these were unsupported by other *in vitro* data, automatically leading to a final rating of “no data” as we were not confident to rate the evidence of an outcome group solely based on gene expression data. This also clearly showed the fact that considerable topic expertise is required to interpret the significance of this type of data. Lastly, there is a need to develop methods to incorporate the level of confidence for “no effect” evidence streams and how these affect the next SYRINA steps as currently, there is little differentiation between “no effects” with low or high confidence. This is especially important in case of contradicting results between different study types (*e.g.* between *in vitro* and *in vivo* data), as in this case study, *in vitro* was given the same weight as *in vivo* endocrine activity data and we did not “downgrade” the strength of evidence *in vitro* due to lack of effects *in vivo.*

### 4.6 Evidence integration across all streams – SYRINA Step 6

Upon determining the strongest evidence in Step 5, we applied the next step of SYRINA to this evidence only as integrating evidence for the whole dataset was determined not to be a suitable approach. However, we provided no clear criteria for what is considered strong enough. Although we used “medium” to “high” evidence based on the plausible link between adversity and endocrine effects, these criteria need to be laid out more precisely. Also, since we found that the link between two streams of evidence is already important in this step, consideration should be given to explore and present these links prior to Step 7. This has been attempted through the OECD Guidance 150,^49^ where the plausible links between adversity and endocrine activity are already to be established for EATS mediated endpoints denoted as EATS mediated endpoints do not need plausible links between adversity and endocrine activity to be established, whereas endpoints “sensitive to but not diagnostic of EATS” require a mode of action analysis. Although the creation of outcome groups partially provides that link in itself, the necessary steps and criteria should be captured and clearly described in the methodology. Furthermore, the matrix presented in Fig. 8 has the potential of overrating *in vitro* information in case of contradicting results with *in vivo* studies. For example, no effects found in an *in vivo* assay will still lead to “strong” evidence if a well-performed *in vitro* study is available. In this case, it is necessary that criteria for weighting of study types is developed in future efforts. In addition to this, criteria need to be established that address the integration of the confidence levels of “no evidence for effect” groups.

### 4.7 Conclusions, recommendations, uncertainties, consequences – SYRINA Step 7

One of the main shortcomings of SYRINA is that a process for how to establish a biologically plausible link between endocrine activity and adverse effect is not described. In this case study, we opted to identify the outcome groups for which there was the strongest evidence and attempted to link those identified effects to a biologically related endocrine activity. This exercise was somewhat hampered by (1) the lack of relevant and reliable *in vivo* data for TPP, even though we chose the substance for its data richness, as well as (2) the fact there is evidence for endocrine activity *via* a number of different endocrine modes of action raises the overall level of concern and suspicion regarding possible ED properties for TPP.

### 4.8 Regulatory context of the framework

Since the SYRINA framework was published in 2016, the EU has implemented scientific criteria and a specific process for identifying EDs within the context of the regulations for plant protection products and biocidal products,^36^ as well as criteria for an ED hazard class in the Classification, Labeling and Packaging (CLP) regulation.^87^ The EU regulatory process and the SYRINA framework are both anchored in the WHO/IPCS definition of an ED, which requires that both endocrine activity and adversity are assessed, as well as establishing a casual link between the endocrine activity and adversity. The EU process for plant protection products and biocidal products also stipulates that a systematic review methodology should be applied. However, there are some differences between the two approaches in regard to systematic searches for and evaluation of information. The EU approach has so far been specifically tailored to the assessment of ED within the regulatory frameworks for plant protection products and biocidal products. This means that there are certain assumptions regarding the availability of data that are based on legislated information requirements for these types of substances, which are relatively substantial. Guidance are in place to help the assessor identify specific data from these data sets that are relevant for the assessment of potential ED properties of plant protection products and biocidal products. However, there is emphasis on also collecting all relevant information from other sources, *e.g.* the published literature and databases, such as ToxCast. This information should be retrieved using systematic review methodologies and subjected to evaluation of reliability and relevance. All evidence should then be integrated using weight of evidence evaluation methods. In effect, the ECHA/EFSA guidance available for the EU process for plant protection products and biocidal products does not describe the different steps of assembling, evaluating and integrating evidence as detailed/strictly as the SYRINA framework. In particular, there is very little guidance for the evaluation of reliability of pieces of evidence or for integrating the evidence in a systematic way, that promotes transparent application of expert judgment. Another major difference between these two processes is that the SYRINA framework allows for the identification of substances as EDs or probable/possible EDs, while the EU process for plant protection products and biocidal products currently only allows the determination of whether a substance is or is not an ED. In the European Commission's Chemicals Strategy for Sustainability towards a Toxic-Free Environment (EC 2020),^88^ EDs are highlighted as being specifically targeted for stricter regulation. This entails, for example, the inclusion of new hazard classes for EDs in the CLP regulation, which requires a weight of evidence assessment to identify known, presumed or suspected EDs. While the SYRINA framework may not be directly applicable for ED identification in the current regulatory context, there are aspects of the framework that can be readily adjusted and used as a basis for further evaluation and identification.

## 5 Conclusion

To conclude, we found that SYRINA can provide a suitable framework for systematically investigating the ED properties of a chemical. As the framework is not restrictive, it is possible to use a variety of approaches and tools within the seven steps of SYRINA. As presented in this study, these tools can be modified in order to fit better in the context of SRs (*e.g.* the SciRAP modifications) and we encourage to explore these possibilities if the stand-alone tool is not completely suitable. Especially in the case of evaluating study quality, criteria that are important for SR purposes should be implemented if missing from the tool. Besides the flexibility of the framework, another important advantage of SYRINA as a systematic review methodology is the structured, well documented, and reproducible means of evaluating information to reach a conclusion.

However, several limitations or challenges were identified and discussed. For example, the inclusion of additional types of literature data other than scientific papers should be considered and the importance of publicly available reports was identified. Furthermore, the requirement of specific scientific expertise for the different types of study for study evaluation, the complexity of handling large amounts of information during evaluation of evidence and therefore the need of diverse expertise were deemed as important and challenging during the course of this work. Another major limitation is the lack of more finding specific reproducible and sensitive criteria for evidence evaluation and integration, are considered some of the major limitations, in addition to the extensive amount of allocated time for evaluation. Furthermore, we would like to acknowledge the need to include an independent evaluator to resolve possible differences in study quality evaluation as this is an important part of an SR, but could not be performed in this work due to capacity limitations.

Based on our conclusions and the challenges of this study, we present the following future research needs:

(1) Test the SYRINA framework on a case with very limited available data in order to investigate how to handle lack of data and subsequent uncertainties.

(2) Explore the possibility and necessity of performing a systematic way of assessment of the links between adverse effects and ED activity. More specifically, an attempt to incorporate adverse outcome pathways into SYRINA in a systematic way while evaluating the strength of the links between adverse effects and ED activity (*e.g.* empirical, mechanistic *etc.*) would contribute to a more cohesive assessment that is consistent with other ED evaluation approaches. The EFSA guidance document for the identification of EDs describes a method to investigate these links and should be carefully taken into consideration. Another option could be to introduce mode of action frameworks from the IPCS (International Programme on Chemical Safety) including application of modified Bradford Hill considerations to the weight of evidence as well as reference to the value of AOPs in establishing biologically plausible links.^89^ The authors describe a quantitative weight of evidence approach to increase transparency and reproducibility for AOP weight of evidence determinations and has the potential to improve the overall confidence in the AOP and this method could be tested for use in the SYRINA framework.

(3) Increase the practicability and reproducibility of the assessment by developing clearer criteria on the selection of data and evidence bodies. This concerns the differentiation between mammalian and non-mammalian data, and the evidence bodies providing the strongest evidence for an ED identification. Furthermore, a method could be developed how to use mammalian or non-mammalian data as supporting information in case the ED assessment is being performed mainly for environmental or human hazard, respectively.

## Abbreviations

TPP: Triphenyl phosphate
ED: Endocrine disruptor
CRA: Chemical risk assessment

## Author contributions

Conceptualization: Thuy T. Bui, Marlene Ågerstrand, and Christina Rudén. Data curation: Thuy T. Bui. Formal analysis: Thuy T. Bui. Funding acquisition: Marlene Ågerstrand and Christina Rudén. Investigation: Thuy T. Bui, Jenny Aasa, Khaled Abass, Marlene Ågerstrand, Anna Beronius, Mafalda Castro, Laura Escrivá, Audrey Galizia, Anda R. Gliga, David Gee, Oskar Karlsson, Paul Whaley, Erin Yost, and Christina Rudén. Methodology: Thuy T. Bui, Anna Beronius, and Paul Whaley. Project administration: Thuy T. Bui, Marlene Ågerstrand, and Christina Rudén. Resources: Christina Rudén. Software: Thuy T. Bui. Supervision: Christina Rudén. Visualization: Thuy T. Bui, Anna Beronius, and Paul Whaley. Writing – original draft: Thuy T. Bui, Anna Beronius, and Paul Whaley. Writing – review & editing: Thuy T. Bui, Jenny Aasa, Khaled Abass, Marlene Ågerstrand, Anna Beronius, Mafalda Castro, Laura Escrivá, Audrey Galizia, Anda R. Gliga, Oskar Karlsson, Paul Whaley, Erin Yost, and Christina Rudén.

## Conflicts of interest

Dr Whaley reports personal fees from Elsevier Ltd (Environment International), the Cancer Prevention and Education Society, the Evidence Based Toxicology Collaboration and Yordas Group, and grants from Lancaster University, which are outside the submitted work but relate to the development and promotion of systematic review and other evidence-based methods in environmental health research, delivering training around these methods, and providing editorial services. The other authors declare no conflict of interest.

## Supplementary Material

EM-026-D3EM00353A-s001

EM-026-D3EM00353A-s002
