# Supplementary material for: Applying a modified systematic review and integrated assessment framework (SYRINA) – a case study on triphenyl phosphate
Source: Environ Sci Process Impacts. 2023 Dec 26;26(2):380–99. doi: 10.1039/d3em00353a (PMC10879963; doi:10.1039/d3em00353a)
Supplement: EM-026-D3EM00353A-s001 [file EM-026-D3EM00353A-s001.pdf]

Electronic Supplementary Material (ESI) for Environmental Science: Processes & Impacts.
This journal is © The Royal Society of Chemistry 2023

Supplemental information 1: Applying a modified systematic review and integrated assessment (SYRINA) framework – A case study on Triphenyl Phosphate

1 Literature search

Search terms for the databases Pubmed, EMBASE, SCOPUS and Web of Science are shown below. The structure was divided into a 1) substance identifier, including synonyms and abbreviations, 2) effect identifier and 3) population identifier according to the PECO statement in Table 1 of the main text. For the population, we used a filter by Koustas et al. (2014) for animal data and a filter from Johnson et al. (2014a) for epidemiological studies. The *in vitro* terms and effect identifiers were constructed with the help of a trained librarian.

PUBMED

("triphenyl phosphate"[tiab] OR "triphenylphosphate"[tiab] OR 115-86-6[rn] OR "TPP" [tiab] OR "TPHP" [tiab])

AND

(developmental biology [mh] OR "development*"[tiab] OR embryonic and fetal development [mh] OR embryo* [tiab] OR fetal [tiab] OR fetus[tiab] OR growth and development [mh] OR growth and development [subheading] OR teratogen*[tiab] OR ecotoxicology [mh] OR ecotoxi* [tiab] OR toxicology [mh] OR toxic* [tiab] OR growth [mh] OR growth* [tiab] OR environment and public health [mh] OR body weight [mh] OR "body weight*" [tiab] OR infant, low birth weight [mh] OR infant, very low birth weight [mh] OR infant, extremely low birth weight [mh] OR embryo loss [mh] OR gestational age [mh] OR gestation* [tiab] OR endocrine system diseases [mh] OR endocrine disruptors [mh] OR endocrine system[mh] OR "endocrine disruptive"[tiab] OR "endocrine disruptor"[tiab] OR "endocrine disruptors"[tiab] OR "endocrine disruption"[tiab] OR hormon*[tiab] OR thyroid diseases [mh] OR thyroid*[tiab] OR thyrox* [tiab]OR androgen antagonists [mh] OR androgen receptor antagonists [mh] OR androgen*[tiab] OR estrogen antagonists [mh] OR estrogen receptor antagonists [mh] OR estrogen*[tiab] OR testosterone [tiab] OR adreno* [tiab] OR adrena* [tiab] OR *corticoid* [tiab] OR cortisol [tiab] OR *cotricotrop* [tiab] OR renin* [tiab] OR angiotensin* [tiab] OR aldosterone* [tiab] OR leptin*[tiab] OR insulin* [tiab] OR ghrelin* [tiab] OR lenomorelin* [tiab] OR reproduction [mh] OR reproduct* [tiab] OR

toxicity [subheading] OR antagonists and inhibitors [subheading] OR Neurotoxicity syndromes [mh] OR neurologic manifestations [mh] OR neurolo* [tiab] OR neurotoxi*[tiab] OR ataxi*[tiab] OR paraly*[tiab] OR neuromoto*[tiab] OR neurodevelop*[tiab] OR neural[tiab] OR Immune system [mh] OR immunology [subheading] OR immunotoxins [mh] OR immunotoxi*[tiab] OR immunolog*[tiab] OR immune* [tiab] OR Metabolism[mh] OR metabolism [subheading] OR metabolic diseases [mh] OR metabolism [tiab] OR metabolic [tiab] OR diabet*[tiab] OR lipid*[tiab] OR obes*[tiab] OR neoplasms [mh] OR carcinogens [mh] OR cancer* [tiab] OR neoplasm* [tiab] OR “peroxisome proliferat*” [tiab] OR carcinogen* [tiab] OR tumor* [tiab] OR tumour* [tiab] OR carcinoma* [tiab] OR leukemi* [tiab])

AND

(toxicity tests [mh] OR "in vitro"[tiab] OR "in-vitro"[tiab] OR "cell*"[tiab] OR “receptor*”[tiab] OR in vitro techniques[Mh] OR cell culture techniques[Mh] OR cells, cultured[Mh]) OR ("in vivo"[tiab] OR “in-vivo”[tiab] "animal experimentation"[Mh] OR "models, animal"[Mh] OR "Animals"[Mesh:noexp] OR "animal population groups"[Mh] OR "chordata"[Mh:noexp] OR "chordata, nonvertebrate"[Mh] OR "vertebrates"[Mh:noexp] OR "amphibians"[Mh] OR "birds"[Mh] OR "fishes"[Mh] OR "reptiles"[Mh] OR "mammals"[Mh:noexp] OR "primates"[Mh:noexp] OR "artiodactyla"[Mh] OR "carnivora"[Mh] OR "cetacea"[Mh] OR "chiroptera"[Mh] OR "elephants"[Mh] OR "hyraxes"[Mh] OR "insectivora"[Mh] OR "lagomorpha"[Mh] OR "marsupialia"[Mh] OR "monotremata"[Mh] OR "perissodactyla"[Mh] OR "rodentia"[Mh] OR "scandentia"[Mh] OR "sirenia"[Mh] OR "xenarthra"[Mh] OR "haplorhini"[Mh:noexp] OR "strepsirhini"[Mh] OR "platyrrhini"[Mh] OR "tarsii"[Mh] OR "catarrhini"[Mh:noexp] OR "cercopithecidae"[Mh] OR "hylobatidae"[Mh] OR "hominidae"[Mh:noexp] OR "gorilla gorilla"[Mh] OR "pan paniscus"[Mh] OR "pan troglodytes"[Mh] OR "pongo pygmaeus"[Mh]) OR (animals[tiab] OR animal[tiab] OR mice[Tiab] OR mus[Tiab] OR mouse[Tiab] OR murine[Tiab] OR woodmouse[tiab] OR rats[Tiab] OR rat[Tiab] OR murinae[Tiab] OR muridae[Tiab] OR cottonrat[tiab] OR cottonrats[tiab] OR hamster[tiab] OR hamsters[tiab] OR cricetinae[tiab] OR rodentia[Tiab] OR rodent[Tiab] OR rodents[Tiab] OR pigs[Tiab] OR pig[Tiab] OR swine[tiab] OR swines[tiab] OR piglets[tiab] OR piglet[tiab] OR boar[tiab] OR boars[tiab] OR "sus scrofa"[tiab] OR ferrets[tiab] OR ferret[tiab] OR polecat[tiab] OR polecats[tiab] OR "mustela putorius"[tiab] OR "guinea pigs"[Tiab] OR "guinea pig"[Tiab] OR cavia[Tiab] OR callithrix[Tiab] OR marmoset[Tiab] OR marmosets[Tiab] OR cebuella[Tiab] OR hapale[Tiab] OR octodon[Tiab] OR chinchilla[Tiab] OR chinchillas[Tiab] OR gerbillinae[Tiab] OR gerbil[Tiab] OR gerbils[Tiab] OR jird[Tiab] OR jirds[Tiab] OR merione[Tiab] OR meriones[Tiab] OR rabbits[Tiab] OR rabbit[Tiab] OR hares[Tiab] OR hare[Tiab] OR diptera[Tiab] OR flies[Tiab] OR fly[Tiab] OR dipteral[Tiab] OR drosphila[Tiab] OR drosophilidae[Tiab] OR cats[Tiab] OR cat[Tiab] OR carus[Tiab] OR felis[Tiab] OR nematoda[Tiab] OR nematode[Tiab] OR nematodes[Tiab] OR sipunculida[Tiab] OR

dogs[Tiab] OR dog[Tiab] OR canine[Tiab] OR canines[Tiab] OR canis[Tiab] OR sheep[Tiab] OR sheeps[Tiab] OR mouflon[Tiab] OR mouflons[Tiab] OR ovis[Tiab] OR goats[Tiab] OR goat[Tiab] OR capra[Tiab] OR capras[Tiab] OR rupicapra[Tiab] OR chamois[Tiab] OR haplorhini[Tiab] OR monkey[Tiab] OR monkeys[Tiab] OR anthropoidea[Tiab] OR anthropoids[Tiab] OR saguinus[Tiab] OR tamarin[Tiab] OR tamarins[Tiab] OR leontopithecus[Tiab] OR hominidae[Tiab] OR ape[Tiab] OR apes[Tiab] OR pan[Tiab] OR paniscus[Tiab] OR "pan paniscus"[Tiab] OR bonobo[Tiab] OR bonobos[Tiab] OR troglodytes[Tiab] OR "pan troglodytes"[Tiab] OR gibbon[Tiab] OR gibbons[Tiab] OR siamang[Tiab] OR siamangs[Tiab] OR nomascus[Tiab] OR symphalangus[Tiab] OR chimpanzee[Tiab] OR chimpanzees[Tiab] OR prosimians[Tiab] OR "bush baby"[Tiab] OR prosimian[Tiab] OR "bush babies"[Tiab] OR galagos[Tiab] OR galago[Tiab] OR pongidae[Tiab] OR gorilla[Tiab] OR gorillas[Tiab] OR pongo[Tiab] OR pygmaeus[Tiab] OR "pongo pygmaeus"[Tiab] OR orangutans[Tiab] OR lemur[Tiab] OR lemurs[Tiab] OR lemuridae[Tiab] OR horse[Tiab] OR horses[Tiab] OR equus[Tiab] OR cow[Tiab] OR calf[Tiab] OR bull[Tiab] OR chicken[Tiab] OR chickens[Tiab] OR gallus[Tiab] OR quail[Tiab] OR bird[Tiab] OR birds[Tiab] OR quails[Tiab] OR poultry[Tiab] OR poultries[Tiab] OR fowl[Tiab] OR fowls[Tiab] OR reptile[Tiab] OR reptilia[Tiab] OR reptiles[Tiab] OR snakes[Tiab] OR snake[Tiab] OR lizard[Tiab] OR lizards[Tiab] OR alligator[Tiab] OR alligators[Tiab] OR crocodile[Tiab] OR crocodiles[Tiab] OR turtle[Tiab] OR turtles[Tiab] OR amphibian[Tiab] OR amphibians[Tiab] OR amphibia[Tiab] OR frog[Tiab] OR frogs[Tiab] OR bombina[Tiab] OR salientia[Tiab] OR toad[Tiab] OR toads[Tiab] OR "epidalea calamita"[Tiab] OR salamander[Tiab] OR salamanders[Tiab] OR eel[Tiab] OR eels[Tiab] OR fish[Tiab] OR fishes[Tiab] OR pisces[Tiab] OR catfish[Tiab] OR catfishes[Tiab] OR siluriformes[Tiab] OR arius[Tiab] OR heteropneustes[Tiab] OR sheatfish[Tiab] OR perch[Tiab] OR perches[Tiab] OR percidae[Tiab] OR perca[Tiab] OR trout[Tiab] OR trouts[Tiab] OR char[Tiab] OR chars[Tiab] OR salvelinus[Tiab] OR "fathead minnow"[Tiab] OR minnow[Tiab] OR cyprinidae[Tiab] OR carps[Tiab] OR carp[Tiab] OR zebrafish[Tiab] OR zebrafishes[Tiab] OR goldfish[Tiab] OR goldfishes[Tiab] OR guppy[Tiab] OR guppies[Tiab] OR chub[Tiab] OR chubs[Tiab] OR tinca[Tiab] OR barbels[Tiab] OR barbus[Tiab] OR pimephales[Tiab] OR promelas[Tiab] OR "poecilia reticulata"[Tiab] OR mullet[Tiab] OR mullets[Tiab] OR seahorse[Tiab] OR seahorses[Tiab] OR "mugil curema"[Tiab] OR "atlantic cod"[Tiab] OR shark[Tiab] OR sharks[Tiab] OR catshark[Tiab] OR anguilla[Tiab] OR salmonid[Tiab] OR salmonids[Tiab] OR whitefish[Tiab] OR whitefishes[Tiab] OR salmon[Tiab] OR salmons[Tiab] OR sole[Tiab] OR solea[Tiab] OR "sea lamprey"[Tiab] OR lamprey[Tiab] OR lampreys[Tiab] OR pumpkinseed[Tiab] OR sunfish[Tiab] OR sunfishes[Tiab] OR tilapia[Tiab] OR tilapias[Tiab] OR turbot[Tiab] OR turbots[Tiab] OR flatfish[Tiab] OR flatfishes[Tiab] OR sciuridae[Tiab] OR squirrel[Tiab] OR squirrels[Tiab] OR chipmunk[Tiab] OR chipmunks[Tiab] OR suslik[Tiab] OR susliks[Tiab] OR vole[Tiab] OR voles[Tiab] OR lemming[Tiab] OR lemmings[Tiab] OR muskrat[Tiab]

OR muskrats[Tiab] OR lemmus[Tiab] OR otter[Tiab] OR otters[Tiab] OR marten[Tiab] OR martens[Tiab] OR martes[Tiab] OR weasel[Tiab] OR badger[Tiab] OR badgers[Tiab] OR ermine[Tiab] OR mink[Tiab] OR minks[Tiab] OR sable[Tiab] OR sables[Tiab] OR gulo[Tiab] OR gulos[Tiab] OR wolverine[Tiab] OR wolverines[Tiab] OR mustela[Tiab] OR llama[Tiab] OR llamas[Tiab] OR alpaca[Tiab] OR alpacas[Tiab] OR camelid[Tiab] OR camelids[Tiab] OR guanaco[Tiab] OR guanacos[Tiab] OR chiroptera[Tiab] OR chiropteras[Tiab] OR bat[Tiab] OR bats[Tiab] OR fox[Tiab] OR foxes[Tiab] OR iguana[Tiab] OR iguanas[Tiab] OR “xenopus laevis”[Tiab] OR parakeet[Tiab] OR parakeets[Tiab] OR parrot[Tiab] OR parrots[Tiab] OR donkey[Tiab] OR donkeys[Tiab] OR mule[Tiab] OR mules[Tiab] OR zebra[Tiab] OR zebras[Tiab] OR shrew[Tiab] OR shrews[Tiab] OR bison[Tiab] OR bisons[Tiab] OR buffalo[Tiab] OR buffaloes[Tiab] OR deer[Tiab] OR deers[Tiab] OR bear[Tiab] OR bears[Tiab] OR panda[Tiab] OR pandas[Tiab] OR "wild hog"[Tiab] OR "wild boar"[Tiab] OR fitchew[Tiab] OR fitch[Tiab] OR beaver[Tiab] OR beavers[Tiab] OR jerboa[Tiab] OR jerboas[Tiab] OR capybara[Tiab] OR capybaras[Tiab]) OR epidemiologic methods [mh] OR epidemiology [mh] OR epidemiolog* [tiab] OR human* [tiab] OR (NOT (animals[mh] NOT humans[mh])))

Web of science

TS=(“triphenyl phosphate” OR “triphenylphosphate” OR TPP OR TPHP)

AND

TS=(teratogen* OR embryo* OR fetal OR fetus OR development* OR ecotoxi* OR toxic* OR growth* OR “body weight*” OR gestation* OR “endocrine disruptive” OR “endocrine disruptor” OR “endocrine disruptors” OR “endocrine disruption” OR hormon* OR thyroid* OR thyrox* OR androgen* OR estrogen* OR testosterone OR adreno* OR adrena* OR *corticoid* OR cortisol OR *cotricotrop* OR renin* OR angiotensin* OR aldosterone* OR leptin* OR insulin* OR ghrelin* OR lenomorelin* OR reproduct* OR neurotoxi* OR ataxi* OR paraly* OR neuromoto* OR neurodevelop* OR neurolo* OR neural OR immunotoxi* OR immunolog* OR immune* OR metabolism OR metabolic OR diabet* OR lipid* OR obes* OR cancer* OR neoplasm* OR “peroxisome proliferat*” OR carcinogen* OR tumor* OR tumour* OR carcinoma* OR leukemi*)

AND

TS=("in vitro" OR "in-vitro" OR "cell*" OR receptor* OR "in vivo" OR in-vivo OR animals OR animal OR mice OR mus OR mouse OR murine OR woodmouse OR rats OR rat OR murinae OR muridae OR

cottonrat OR cottonrats OR hamster OR hamsters OR cricetinae OR rodentia OR rodent OR rodents OR pigs OR pig OR swine OR swines OR piglets OR piglet OR boar OR boars OR "sus scrofa" OR ferrets OR ferret OR polecat OR polecats OR "mustela putorius" OR "guinea pigs" OR "guinea pig" OR cavia OR callithrix OR marmoset OR marmosets OR cebuella OR hapale OR octodon OR chinchilla OR chinchillas OR gerbillinae OR gerbil OR gerbils OR jird OR jirds OR merione OR meriones OR rabbits OR rabbit OR hares OR hare OR diptera OR flies OR fly OR dipteral OR drosphila OR drosophilidae OR cats OR cat OR carus OR felis OR nematoda OR nematode OR nematodes OR sipunculida OR dogs OR dog OR canine OR canines OR canis OR sheep OR sheeps OR mouflon OR mouflons OR ovis OR goats OR goat OR capra OR capras OR rupicapra OR chamois OR haplorhini OR monkey OR monkeys OR anthropoidea OR anthropoids OR saguinus OR tamarin OR tamarins OR leontopithecus OR hominidae OR ape OR apes OR pan OR paniscus OR "pan paniscus" OR bonobo OR bonobos OR troglodytes OR "pan troglodytes" OR gibbon OR gibbons OR siamang OR siamangs OR nomascus OR symphalangus OR chimpanzee OR chimpanzees OR prosimians OR "bush baby" OR prosimian OR bush babies OR galagos OR galago OR pongidae OR gorilla OR gorillas OR pongo OR pygmaeus OR "pongo pygmaeus" OR orangutans OR lemur OR lemurs OR lemuridae OR horse OR horses OR equus OR cow OR calf OR bull OR chicken OR chickens OR gallus OR quail OR bird OR birds OR quails OR poultry OR poultries OR fowl OR fowls OR reptile OR reptilia OR reptiles OR snakes OR snake OR lizard OR lizards OR alligator OR alligators OR crocodile OR crocodiles OR turtle OR turtles OR amphibian OR amphibians OR amphibia OR frog OR frogs OR bombina OR salientia OR toad OR toads OR "epidalea calamita" OR salamander OR salamanders OR eel OR eels OR fish OR fishes OR pisces OR catfish OR catfishes OR siluriformes OR arius OR heteropneustes OR sheatfish OR perch OR perches OR percidae OR perca OR trout OR trouts OR char OR chars OR salvelinus OR "fathead minnow" OR minnow OR cyprinidae OR carps OR carp OR zebrafish OR zebrafishes OR goldfish OR goldfishes OR guppy OR guppies OR chub OR chubs OR tinca OR barbels OR barbus OR pimephales OR promelas OR "poecilia reticulata" OR mullet OR mullets OR seahorse OR seahorses OR “mugil curema” OR “atlantic cod” OR shark OR sharks OR catshark OR anguilla OR salmonid OR salmonids OR whitefish OR whitefishes OR salmon OR salmons OR sole OR solea OR "sea lamprey" OR lamprey OR lampreys OR pumpkinseed OR sunfish OR sunfishes OR tilapia OR tilapias OR turbot OR turbots OR flatfish OR flatfishes OR sciuridae OR squirrel OR squirrels OR chipmunk OR chipmunks OR suslik OR susliks OR vole OR voles OR lemming OR lemmings OR muskrat OR muskrats OR lemmus OR otter OR otters OR marten OR martens OR martes OR weasel OR badger OR badgers OR ermine OR mink OR minks OR sable OR sables OR gulo OR gulos OR wolverine OR wolverines OR mustela OR llama OR llamas OR alpaca OR alpacas OR camelid OR camelids OR guanaco OR guanacos OR chiroptera OR chiropteras OR bat OR bats OR fox OR foxes OR iguana OR iguanas OR “xenopus laevis” OR parakeet OR parakeets OR parrot OR parrots OR donkey OR donkeys OR mule OR mules OR

zebra OR zebras OR shrew OR shrews OR bison OR bisons OR buffalo OR buffaloes OR deer OR deers OR bear OR bears OR panda OR pandas OR "wild hog" OR "wild boar" OR fitchew OR fitch OR beaver OR beavers OR jerboa OR jerboas OR capybara OR capybaras OR epidemiolog* OR human*)

SCOPUS

(TITLE-ABS-KEY("triphenyl phosphate" OR "triphenylphosphate" OR "TPP" OR "TPHP") OR CASREGNUMBER(115-86-6))

AND

(TITLE-ABS-KEY(teratogen* OR embryo* OR fetal OR fetus OR development* OR ecotoxi* OR toxic* OR growth* OR “body weight*” OR gestation* OR “endocrine disruptive” OR “endocrine disruptor” OR “endocrine disruptors” OR “endocrine disruption” OR hormon* OR thyroid* OR thyrox* OR androgen* OR estrogen* OR testosterone OR adreno* OR adrena* OR *corticoid* OR cortisol OR *cotricotrop* OR renin* OR angiotensin* OR aldosterone* OR leptin* OR insulin* OR ghrelin* OR lenomorelin* OR reproduct* OR neurotoxi* OR ataxi* OR paraly* OR neuromoto* OR neurodevelop* OR neurolo* OR neural OR immunotoxi* OR immunolog* OR immune* OR metabolism OR metabolic OR diabet* OR lipid* OR obes* OR cancer* OR neoplasm* OR “peroxisome proliferat*” OR carcinogen* OR tumor* OR tumour* OR carcinoma* OR leukemi*))

AND

(TITLE-ABS-KEY("in vitro" OR "in-vitro" OR "cell*" OR receptor* OR "in vivo" OR in-vivo OR animals OR animal OR mice OR mus OR mouse OR murine OR woodmouse OR rats OR rat OR murinae OR muridae OR cottonrat OR cottonrats OR hamster OR hamsters OR cricetinae OR rodentia OR rodent OR rodents OR pigs OR pig OR swine OR swines OR piglets OR piglet OR boar OR boars OR "sus scrofa" OR ferrets OR ferret OR polecat OR polecats OR "mustela putorius" OR "guinea pigs" OR "guinea pig" OR cavia OR callithrix OR marmoset OR marmosets OR cebuella OR hapale OR octodon OR chinchilla OR chinchillas OR gerbillinae OR gerbil OR gerbils OR jird OR jirds OR merione OR meriones OR rabbits OR rabbit OR hares OR hare OR diptera OR flies OR fly OR dipteral OR drosphila OR drosophilidae OR cats OR cat OR carus OR felis OR nematoda OR nematode OR nematodes OR sipunculida OR dogs OR dog OR canine OR canines OR canis OR sheep OR sheeps OR mouflon OR mouflons OR ovis OR goats OR goat OR capra OR capras OR rupicapra OR chamois OR haplorhini OR monkey OR monkeys OR anthropoidea OR anthropoids OR saguinus OR tamarin OR tamarins OR leontopithecus OR hominidae OR

ape OR apes OR pan OR paniscus OR "pan paniscus" OR bonobo OR bonobos OR troglodytes OR "pan troglodytes" OR gibbon OR gibbons OR siamang OR siamangs OR nomascus OR symphalangus OR chimpanzee OR chimpanzees OR prosimians OR "bush baby" OR prosimian OR “bush babies” OR galagos OR galago OR pongidae OR gorilla OR gorillas OR pongo OR pygmaeus OR "pongo pygmaeus" OR orangutans OR lemur OR lemurs OR lemuridae OR horse OR horses OR equus OR cow OR calf OR bull OR chicken OR chickens OR gallus OR quail OR bird OR birds OR quails OR poultry OR poultries OR fowl OR fowls OR reptile OR reptilia OR reptiles OR snakes OR snake OR lizard OR lizards OR alligator OR alligators OR crocodile OR crocodiles OR turtle OR turtles OR amphibian OR amphibians OR amphibia OR frog OR frogs OR bombina OR salientia OR toad OR toads OR "epidalea calamita" OR salamander OR salamanders OR eel OR eels OR fish OR fishes OR pisces OR catfish OR catfishes OR siluriformes OR arius OR heteropneustes OR sheatfish OR perch OR perches OR percidae OR perca OR trout OR trouts OR char OR chars OR salvelinus OR "fathead minnow" OR minnow OR cyprinidae OR carps OR carp OR zebrafish OR zebrafishes OR goldfish OR goldfishes OR guppy OR guppies OR chub OR chubs OR tinca OR barbels OR barbus OR pimephales OR promelas OR "poecilia reticulata" OR mullet OR mullets OR seahorse OR seahorses OR “mugil curema” OR “atlantic cod” OR shark OR sharks OR catshark OR anguilla OR salmonid OR salmonids OR whitefish OR whitefishes OR salmon OR salmons OR sole OR solea OR "sea lamprey" OR lamprey OR lampreys OR pumpkinseed OR sunfish OR sunfishes OR tilapia OR tilapias OR turbot OR turbots OR flatfish OR flatfishes OR sciuridae OR squirrel OR squirrels OR chipmunk OR chipmunks OR suslik OR susliks OR vole OR voles OR lemming OR lemmings OR muskrat OR muskrats OR lemmus OR otter OR otters OR marten OR martens OR martes OR weasel OR badger OR badgers OR ermine OR mink OR minks OR sable OR sables OR gulo OR gulos OR wolverine OR wolverines OR mustela OR llama OR llamas OR alpaca OR alpacas OR camelid OR camelids OR guanaco OR guanacos OR chiroptera OR chiropteras OR bat OR bats OR fox OR foxes OR iguana OR iguanas OR “xenopus laevis” OR parakeet OR parakeets OR parrot OR parrots OR donkey OR donkeys OR mule OR mules OR zebra OR zebras OR shrew OR shrews OR bison OR bisons OR buffalo OR buffaloes OR deer OR deers OR bear OR bears OR panda OR pandas OR "wild hog" OR "wild boar" OR fitchew OR fitch OR beaver OR beavers OR jerboa OR jerboas OR capybara OR capybaras OR epidemiolog* OR human*))

EMBASE

((‘triphenyl phosphate’ OR triphenylphosphate OR TPP OR TPHP):ti,ab)

AND

(health/exp OR cardiotoxicity/exp OR ‘chronic toxicity’/exp OR ‘drug toxicity’/exp OR ecotoxicity/exp OR embryotoxicity/exp OR ‘experimental toxicity’/exp OR fetotoxicity/exp OR genotoxicity/exp OR ‘liver toxicity’/exp OR teratogenicity/exp OR ‘body weight’/exp OR reproduction/exp OR ‘reproductive success’/exp OR ‘reproductive toxicity’/exp OR ‘reproductive health’/exp OR ‘delayed neurotoxicity’/exp OR ‘neurologic disease’/exp OR neurotoxicity/exp OR immunotoxicity/exp OR ‘metabolic disorder’/exp OR glucotoxicity/exp OR lipotoxicity/exp OR neoplasm/exp (teratogen* OR embryo* OR fetal OR fetus OR development* OR ecotoxi* OR toxic* OR growth* OR ‘body weight*’ OR gestation* OR ‘endocrine disruptive’ OR ‘endocrine disruptor’ OR ‘endocrine disruptors’ OR ‘endocrine disruption’ OR hormon* OR thyroid* OR thyrox* OR androgen* OR estrogen* OR testosterone OR adreno* OR adrena* OR corticoid* OR cortisol OR cotricotrop* OR renin* OR angiotensin* OR aldosterone* OR leptin* OR insulin* OR ghrelin* OR lenomorelin* OR reproduct* OR neurotoxi* OR ataxi* OR paraly* OR neuromoto* OR neurodevelop* OR neurolo* OR neural OR immunotoxi* OR immunolog* OR immune* OR metabolism OR metabolic OR diabet* OR lipid* OR obes* OR cancer* OR neoplasm* OR ‘peroxisome proliferat*’ OR carcinogen* OR tumor* OR tumour* OR carcinoma* OR leukemi*):ti,ab)

AND

((‘in vitro’ OR ‘in-vitro’ OR ‘cell*’ OR receptor* OR ‘in vivo’ OR ‘in-vivo’ OR animals OR animal OR mice OR mus OR mouse OR murine OR woodmouse OR rats OR rat OR murinae OR muridae OR cottonrat OR cottonrats OR hamster OR hamsters OR cricetinae OR rodentia OR rodent OR rodents OR pigs OR pig OR swine OR swines OR piglets OR piglet OR boar OR boars OR ‘sus scrofa’ OR ferrets OR ferret OR polecat OR polecats OR ‘mustela putorius’ OR ‘guinea pigs’ OR ‘guinea pig’ OR cavia OR callithrix OR marmoset OR marmosets OR cebuella OR hapale OR octodon OR chinchilla OR chinchillas OR gerbillinae OR gerbil OR gerbils OR jird OR jirds OR merione OR meriones OR rabbits OR rabbit OR hares OR hare OR diptera OR flies OR fly OR dipteral OR drosphila OR drosophilidae OR cats OR cat OR carus OR felis OR nematoda OR nematode OR nematodes OR sipunculida OR dogs OR dog OR canine OR canines OR canis OR sheep OR sheeps OR mouflon OR mouflons OR ovis OR goats OR goat OR capra OR capras OR rupicapra OR chamois OR haplorhini OR monkey OR monkeys OR anthropoidea OR anthropoids OR saguinus OR tamarin OR tamarins OR leontopithecus OR hominidae OR ape OR apes OR pan OR paniscus OR ‘pan paniscus’ OR bonobo OR bonobos OR troglodytes OR ‘pan troglodytes’ OR gibbon OR gibbons OR siamang OR siamangs OR nomascus OR symphalangus OR chimpanzee OR chimpanzees OR prosimians OR ‘bush baby’ OR prosimian OR ‘bush babies’ OR galagos OR galago OR pongidae OR gorilla OR gorillas OR pongo OR pygmaeus OR ‘pongo pygmaeus’ OR orangutans OR lemur OR lemurs OR lemuridae OR horse OR horses OR equus OR cow OR calf OR bull OR chicken OR chickens OR gallus OR quail OR bird OR birds OR quails OR poultry OR poultries OR fowl OR fowls OR reptile OR reptilia

OR reptiles OR snakes OR snake OR lizard OR lizards OR alligator OR alligators OR crocodile OR crocodiles OR turtle OR turtles OR amphibian OR amphibians OR amphibia OR frog OR frogs OR bombina OR salientia OR toad OR toads OR 'epidalea calamita' OR salamander OR salamanders OR eel OR eels OR fish OR fishes OR pisces OR catfish OR catfishes OR siluriformes OR arius OR heteropneustes OR sheatfish OR perch OR perches OR percidae OR perca OR trout OR trouts OR char OR chars OR salvelinus OR 'fathead minnow' OR minnow OR cyprinidae OR carps OR carp OR zebrafish OR zebrafishes OR goldfish OR goldfishes OR guppy OR guppies OR chub OR chubs OR tinca OR barbels OR barbus OR pimephales OR promelas OR 'poecilia reticulata' OR mullet OR mullets OR seahorse OR seahorses OR 'mugil curema' OR 'atlantic cod' OR shark OR sharks OR catshark OR anguilla OR salmonid OR salmonids OR whitefish OR whitefishes OR salmon OR salmons OR sole OR solea OR 'sea lamprey' OR lamprey OR lampreys OR pumpkinseed OR sunfish OR sunfishes OR tilapia OR tilapias OR turbot OR turbots OR flatfish OR flatfishes OR sciuridae OR squirrel OR squirrels OR chipmunk OR chipmunks OR suslik OR susliks OR vole OR voles OR lemming OR lemmings OR muskrat OR muskrats OR lemmus OR otter OR otters OR marten OR martens OR martes OR weasel OR badger OR badgers OR ermine OR mink OR minks OR sable OR sables OR gulo OR gulos OR wolverine OR wolverines OR mustela OR llama OR llamas OR alpaca OR alpacas OR camelid OR camelids OR guanaco OR guanacos OR chiroptera OR chiropteras OR bat OR bats OR fox OR foxes OR iguana OR iguanas OR 'xenopus laevis' OR parakeet OR parakeets OR parrot OR parrots OR donkey OR donkeys OR mule OR mules OR zebra OR zebras OR shrew OR shrews OR bison OR bisons OR buffalo OR buffaloes OR deer OR deers OR bear OR bears OR panda OR pandas OR 'wild hog' OR 'wild boar' OR fitchew OR fitch OR beaver OR beavers OR jerboa OR jerboas OR capybara OR capybaras OR epidemiolog* OR human*):ti,ab)

2 Study evaluation

2.1 SciRAP criteria for *in vivo* and *in vitro* studies

SciRAP tools for the evaluation of both *in vivo* animal toxicity studies as well as *in vitro* studies consist of several criteria, divided into reporting quality and methodological quality (Table SI-1). For use in the context of SRs, we do not use criteria for relevance, which would usually be a part of the tool. The reason for not including a relevance criteria is that these study were already screened (abstract and full text) for relevance according to criteria in Step 3b. Also, we modified the SciRAP tools in order to cover all relevant risk of bias domains used in traditional SRs. To be more specific, we included additional criteria for blinding and attrition, which were not a part of SciRAP initially. Blinding and attrition are considered important criteria in order to minimize bias due to knowing treatment groups beforehand or omitting results from the final analysis, respectively. Each criterion can be evaluated as "fulfilled", "partially fulfilled" or "not

fulfilled" (translating to "good", "deficient" and "critically deficient" on the HAWC platform). The tool also provides guidance on how to judge each criterion in the methodological quality segment. We added an "overall confidence rating" criterion for both tools to be able to arrive to a final evaluation score similarly to other risk of bias tools. This decision is made by expert judgment considering all prior aspects. Guidance text and instructions how to judge the criteria can be viewed on the draft of the publicly available assessment (https://hawcprd.epa.gov/assessment/100000054/). For the rating of the overall study confidence we used 4 ratings "high", "medium", "low" and "uninformative". Uninformative studies were not be considered in further analysis.

Table SI-1 Criteria used in the evaluation of individual studies for animal toxicity and *in vitro* mechanistic studies. Detailed guidance and instructions on how to judge the methodological criteria can be viewed at https://hawcprd.epa.gov/assessment/100000054/.

**Criterion name**	**Description**	**Animal toxicity**	***In vitro* mechanistic**
	**Reporting quality**		
Chemical name	The chemical name, ID or CAS-number of the test compound was given.	x	x
Purity of compound	The purity of the test compound was stated or is traceable according to information given regarding manufacturer and lot/batch number. In case of mixtures, the composition of different constituents was stated.	x	x
Solubility of the compound	The solubility of the test compound was described.		x
Vehicle	The vehicle was described.	x	x
Control group	It was stated that a negative control / untreated group / vehicle control was included.	x	x
Test system	The test system (cell line / cells/ tissue / organ / embryo) was described.		x
Source of test system	The source of the test system was stated.		x
Metabolic competence	Metabolic competence of the test system was described.		x
Number of cell passages	The number of cell passages of the cell line used, was stated. (rate with 'good' if no cell lines were used and include a comment)		x
Media composition	Composition of media was described, including use of serum, antibiotics, etc.		x
Incubation parameters	Incubation temperature, humidity, and CO2 concentration were described.		x
Contamination	Measures taken for avoiding or screening for contamination by mycoplasma, bacteria, fungi and virus were described.		x
Cell density	Cell density or number of cells used during treatment was		x

	described. (Remove this criterion if the study was not conducted in a cell line.)		
Treatment duration	The duration of treatment was stated.		x
Replicates/repetitions	The number of replicates per dose level/concentration or the number of times the experiment was repeated was stated.		x
Animal model	The animal model (species, strain, age or life stage and sex) was described.	x	
Individual identification	The method for individual identification of animals was described.	x	
Housing temperature	The housing temperature was stated.	x	
Relative humidity	The relative humidity was stated.	x	
Light-dark cycle	The light-dark cycle was described.	x	
Number of animals	The number of animals per sex in each cage was stated.	x	
Cage material	The cage materials were described.	x	
Physical enrichment	Any materials used for physical enrichment were described.	x	
Water bottle material	Water bottle materials were described.	x	
Bedding material	The bedding material used was described.	x	
Feed	The type and source of feed were reported.	x	
Drinking water	The source of drinking water was reported.	x	
Dose levels	The administered dose levels or concentrations were stated.	x	x
Allocation	The method for allocating animals to different treatments was stated.	x	
Number of animals in dose groups	The total number of animals per dose group was stated.	x	
Route of administration	The route of administration was stated.	x	
Sex and age of animals at dosing	The sex and age (or life stage) of the animals at start of dosing was stated or is obvious from the information given, e.g. "pregnant rats were used" is enough information that animals are female and sexually mature/adult.	x	
Frequency and duration of dosing	The frequency and duration of dosing/administration of the test compound was stated.	x	
Test/analytical methods	The tests and/or analytical methods used were sufficiently described to allow for evaluation of reliability of results.	x	x
Data collection time-points	The time points for data collection were stated.		x
Cytotoxicity	It was stated that the effect of the test compound on cytotoxicity was measured.		x
Allocation to different tests	The method for allocating animals to different tests and measurements (e.g. tissue collection or evaluation of functional or behavioral endpoints) was stated.	x	
Animals subjected to separate tests	The sex, age and number of animals per dose group subjected to separate tests and measurements was stated.	x	
Results presentation	All results for the investigated endpoints were clearly reported. The most critical results were presented in tables and figures, including description of variation and	x	x

	statistically significant results.		
Statistical methods and software	The statistical methods and software used were described.	x	x
Statistical unit	The statistical unit, e.g. the individual or the litter, was stated.	x	
Funding	The funding sources for the study were stated.	x	x
Competing interests	Any competing interests were disclosed or it was explicitly stated that the authors did not have any competing interests.	x	x
**Methodological quality**			
Purity	The test compound or mixture was unlikely to contain any impurities that may significantly have affected the results of the study.	x	x
Solubility of the compound	It was likely that the test compound was soluble at the concentrations used.		x
Vehicle	An appropriate vehicle was used that is not expected to interfere with the absorption, distribution, metabolism, excretion or toxicity of the test compound.	x	x
Negative control group	A concurrent negative control group was included.	x	
Untreated/vehicle control	An untreated or vehicle control was included.		x
Test system	A reliable and sensitive test system (cell line / cells / tissue / organ /embryo) with metabolic competence, if relevant, was used for investigating the test compound and endpoints.		x
Conditions for cultivation	Conditions for cultivation and/or maintenance of the cell line / cells / tissue / organ /embryo (incubation temperature, humidity, CO2 concentration, media used, number of cell passages, control of contamination) were appropriate.		x
Animal model	A reliable and sensitive animal model was used for investigating the test compound and selected endpoints.	x	
Individual identification	Animals were individually identified.	x	
Housing conditions	Housing conditions (temperature, relative humidity, light-dark cycle) were appropriate for the study type and animal model.	x	
Number of animals in cage	The number of animals per sex in each cage were appropriate for the study type and animal model.	x	
Contaminants	The test system was unlikely to contain contaminants that could affect study results, such as organic pollutants, pesticide residues, heavy metals, and mycotoxins, as well as phytoestrogens.	x	
Allocation to treatments	The allocation of animals to different treatments was randomized.	x	
Route of administration	The route of administration was appropriate and not likely to interfere with the study results.	x	
Timing and duration of administration	The timing and duration of administration were appropriate for investigating the included endpoints.	x	

Duration of exposure	The duration of exposure was suitable for the test system and investigated endpoints.		x
Concentrations used	The concentrations used were suitable for the test system and investigated endpoints.		x
Test conditions	The test conditions during and after exposure to the test compound were suitable (media and serum used, cell density, incubation temperature, humidity, CO_2 concentration).		x
Frequency of administration	The frequency of administration was appropriate for investigating the included endpoints.	x	
Allocation to different tests	The allocation of animals to different tests and measurements was randomized.	x	
Test methods	Reliable and sensitive test methods were used for investigating the selected endpoints.	x	x
Collection of measurements	Measurements were collected at suitable time points in order to generate sensitive, valid and reliable data.	x	
Replicates/repetitions	Sufficient numbers of replicates or repetitions of the experiment were used to generate reliable and valid results.		x
Cytotoxicity	Cytotoxicity was measured and the test compound did not cause cytotoxicity that significantly affected the results.		x
Number of animals	A sufficient number of animals per dose group were subjected to separate tests/data collection/measurements to generate reliable and valid results.	x	
Statistical methods and software	The statistical methods were clearly described and do not seem inappropriate, unusual or unfamiliar.	x	x
Attrition	Did the study report results for all tested animals/replicates/repetitions?	x	x
Blinding or similar measures	Did the study implement measures to reduce observational bias?	x	x
Overall study confidence	No description	x	x

2.2 IRIS domains for epidemiological studies

Table SI-2 Domains used in the evaluation of individual studies for human epidemiological studies. Detailed guidance, instructions and examples can be viewed at https://hawcprd.epa.gov/assessment/100000054/.

**Domain**	**Core question**
Selection and Performance	Is there evidence that selection into or out of the study (or analysis sample) was jointly related to exposure and to outcome?
Exposure methods	Does the exposure measure reliably distinguish between levels of exposure in a time window considered most relevant for a causal effect with respect to the development of the outcome?
Outcome methods/results presentation	Does the outcome measure reliably distinguish the presence or absence (or degree of severity) of the outcome?

Confounding	Is confounding of the effect of the exposure unlikely?
Analysis	Does the analysis strategy and presentation convey the necessary familiarity with the data and assumptions?
Selective reporting	Is there concern for selective reporting?
Sensitivity	Are there concerns for study sensitivity?
Overall confidence	No core question

2.3 Study evaluation results

Table SI-3 Excluded studies after study quality evaluation.

Study	Title	Type	Reasoning
Ahrens et al. (1978)	A Water-extractable Toxic Compound In Vinyl Upholstery Fabric	Animal bioassay	**Uninformative**<br>Generally poor reporting and results presentation<br><br>Methods are poorly described; criteria for evaluation are unclear;, no statistics are given.
Theiss et al. (1977)	Test for Carcinogenicity of Organic Contaminants of United States Drinking Waters by Pulmonary Tumor Response in Strain A Mice	Animal bioassay	**Uninformative**<br>Despite being a brief communication and therefore, poorly reported, the study also shows major flaws in exposure duration, number of animals used, and endpoints investigated. This study has serious flaws, primarily in regard to reporting, lack of randomization and blinding procedures, and potential attrition bias. It is also not clear if the number of adenomas are only counted in surviving animals or if animals lost during treatment are also included in analyses.
Wills et al. (1979)	Does Triphenyl Phosphate Produce Delayed Neurotoxic Effects?	Animal bioassay	**Uninformative**<br>Many flaws and very small study that is briefly described
Fautz et al. (1993)	Immunotoxicity screening in vitro using an economical multiple endpoint approach	*in vitro*	**Uninformative**<br>General: purity and vehicle not clear, incubation parameters poorly reported, unclear what kind of controls were used, no quantitative display of results for TPP; the only useful information is Table 1.However considering the other major flaws, this study is clearly uninformative.
Obersteiner et al.	Evaluation of Cytotoxic Responses Caused by	*in vitro*	**Uninformative**<br>Very poor reporting, no detailed results for TPP

(1978)	Selected Organophosphorus Esters in Chick Sympathetic Ganglia Cultures		shown, evaluation not blinded although it could be influenced significantly
Scanlan et al. (2015)	Gene Transcription, Metabolite and Lipid Profiling in Eco-Indicator Daphnia magna Indicate Diverse Mechanisms of Toxicity by Legacy and Emerging Flame-Retardants	*in vitro*	**Uninformative**<br>Although the study was well conducted, it only investigated acute toxicity of TPP on *Daphnia magna* and is therefore not relevant to the review question.
Pei et al. (2016)	Comparative neurotoxicity screening in human iPSC-derived neural stem cells, neurons and astrocytes	*in vitro*	**Uninformative**<br>The study only investigated cytotoxicity which is too unspecific for endocrine disruption. Therefore, the study was judged to be uninformative.

References

Ahrens, V.D., Henion, J.D., Maylin, G.A., Leibovitz, L., St John, L.E., Lisk, D.J., 1978. A water-extractable toxic compound in vinyl upholstery fabric. Bull. Environ. Contam. Toxicol. 20, 418–422.

Beronius, A., Ågerstrand, M., 2017. Making the most of expert judgment in hazard and risk assessment of chemicals. Toxicol. Res. (Camb). 6, 571–577. doi:10.1039/c7tx00114b

Fautz, R., Miltenburger, H.G., 1993. Immunotoxicity screening in vitro using an economical multiple endpoint approach. Toxicol. Vitr. 7, 305–310. doi:10.1016/0887-2333(93)90019-2

Ingre-Khans, E., Ågerstrand, M., Rudén, C., Beronius, A., 2019. Improving structure and transparency in reliability evaluations of data under REACH: suggestions for a systematic method. Hum. Ecol. Risk Assess. 1–30. doi:10.1080/10807039.2018.1504275

Johnson, P.I., Sutton, P., Atchley, D.S., Koustas, E., Lam, J., Sen, S., Robinson, K.A., Axelrad, D.A., Woodruff, T.J., 2014a. The Navigation guide—evidence-based medicine meets environmental health: Systematic review of human evidence for PFOA effects on fetal growth. Environ. Health Perspect. doi:10.1289/ehp.1307893

Johnson, P.I., Sutton, P., Atchley, D.S., Koustas, E., Lam, J., Sen, S., Robinson, K.A., Axelrad, D.A., Woodruff, T.J., 2014b. The Navigation guide—evidence-based medicine meets environmental health: Systematic review of human evidence for PFOA effects on fetal growth. Environ. Health Perspect. doi:10.1289/ehp.1307893

Koustas, E., Lam, J., Sutton, P., Johnson, P.I., Atchley, D.S., Sen, S., Robinson, K.A., Axelrad, D.A., Woodruff, T.J., 2014. The Navigation Guide - evidence-based medicine meets environmental health: systematic review of nonhuman evidence for PFOA effects on fetal growth. Environ. Health Perspect. 122, 1015–27. doi:10.1289/ehp.1307177

Molander, L., Ågerstrand, M., Beronius, A., Hanberg, A., Rudén, C., 2015. Science in Risk Assessment and Policy (SciRAP): An Online Resource for Evaluating and Reporting In Vivo (Eco)Toxicity

Studies. Hum. Ecol. Risk Assess. 21, 753–762. doi:10.1080/10807039.2014.928104

Obersteiner, E.J., Sharma, R.P., 1978. Evaluation of Cytotoxic Responses Caused by Selected Organophosphorus Esters in Chick Sympathetic Ganglia Cultures. Can J Comp Med 42, 80–88.

Pei, Y., Peng, J., Behl, M., Sipes, N.S., Shockley, K.R., Rao, M.S., Tice, R.R., Zeng, X., 2016. Comparative neurotoxicity screening in human iPSC-derived neural stem cells, neurons and astrocytes. Brain Res. 1638, 57–73. doi:10.1016/j.brainres.2015.07.048

Scanlan, L.D., Loguinov, A. V., Teng, Q., Antczak, P., Dailey, K.P., Nowinski, D.T., Kornbluh, J., Lin, X.X., Lachenauer, E., Arai, A., Douglas, N.K., Falciani, F., Stapleton, H.M., Vulpe, C.D., 2015. Gene Transcription, Metabolite and Lipid Profiling in Eco-Indicator Daphnia magna Indicate Diverse Mechanisms of Toxicity by Legacy and Emerging Flame-Retardants. Environ. Sci. Technol. 49, 7400–7410. doi:10.1021/acs.est.5b00977

Theiss, J.C., Shimkin, M.B., Weisburger, E.K., 1977. Test for Carcinogenicity of Organic Contaminants of United States Drinking Waters by Pulmonary Tumor Response in Strain A Mice. Cancer Res. 37, 2717–2720.

Wills, J.H., Barron, K., Groblewski, G.E., Benitz, K.F., Johnson, M.K., 1979. Does triphenyl phosphate produce delayed neurotoxic effects? Toxicol. Lett. 4, 21–24. doi:10.1016/0378-4274(79)90025-0

Yost, E.E., Euling, S.Y., Weaver, J.A., Beverly, B.E.J., Keshava, N., Mudipalli, A., Arzuaga, X., Blessinger, T., Dishaw, L., Hotchkiss, A., Makris, S.L., 2018. Hazards of diisobutyl phthalate (DIBP) exposure: A systematic review of animal toxicology studies. Environ. Int. 125, 579–594. doi:10.1016/j.envint.2018.09.038
